# Recruitment variation disrupts the stability of alternative life histories in an exploited salmon population

**DOI:** 10.1111/eva.12709

**Published:** 2018-12-01

**Authors:** Lukas B. DeFilippo, Daniel E. Schindler, Jan Ohlberger, Kevin L. Schaberg, Matt Birch Foster, Darin Ruhl, André E. Punt

**Affiliations:** ^1^ School of Aquatic and Fishery Sciences University of Washington Seattle Washington; ^2^ Alaska Department of Fish and Game Division of Commercial Fisheries Westward Region Office Kodiak Alaska

**Keywords:** alternative reproductive tactic, Bayesian state‐space, cohort mismatch, fisheries‐induced evolution, frequency‐dependent selection, jack, recruitment, sneaker male

## Abstract

Males of many fish species exhibit alternative reproductive tactics, which can influence the maturation schedules, fishery productivity, and resilience to harvest of exploited populations. While alternative mating phenotypes can persist in stable equilibria through frequency‐dependent selection, shifts in tactic frequencies have been observed and can have substantial consequences for fisheries. Here, we examine the dynamics of precocious sneaker males called “jacks” in a population of sockeye salmon (*Oncorhynchus nerka*) from Frazer Lake, Alaska. Jacks, which are of little commercial value due to their small body sizes, have recently been observed at unusually high levels in this stock, degrading the value of regional fisheries. To inform future strategies for managing the prevalence of jacks, we used long‐term monitoring data to identify what regulates the frequencies of alternative male phenotypes in the population over time. Expression of the jack life history could not be explained by environmental factors expected to influence juvenile body condition and maturation probability. Instead, we found a strong positive association between the proportion of individuals maturing as jacks within a cohort and the prevalence of jacks among the males that sired that cohort. Moreover, due to differences in age‐at‐maturity between male phenotypes, and large interannual variability in recruitment strength, jacks from strong year‐classes often spawn among older males from the weaker recruitments of earlier cohorts. Through such “cohort mismatches,” which are amplified by size‐selective harvest on older males, jacks frequently achieve substantial representation in the breeding population, and likely high total fertilizations. The repeated occurrence of these cohort mismatches appears to disrupt the stabilizing influence of frequency‐dependent selection, allowing the prevalence of jacks to exceed what might be expected under equilibrium conditions. These results emphasize that the dynamics of alternative life histories can profoundly influence fishery performance and should be explicitly considered in the management of exploited populations.

## 1 INTRODUCTION

There is increasing awareness that fisheries management must consider the mating systems of exploited taxa to understand the biological consequences of exploitation (reviewed in Rowe & Hutchings, 2003). In fishes, males of the same species commonly exhibit divergent approaches to achieving fertilizations, which can influence the genetic diversity, age and size structure, and fishery productivity of exploited stocks (Jones & Hutchings, 2002; Larsen et al., 2004; Myers, 1984; Rowe & Hutchings, 2003). Reflecting discontinuous variation in one or more traits within a population, the existence of such alternative reproductive tactics (ARTs, sensu Taborsky, Oliveira, & Brockmann, 2008) challenged early evolutionary models based on optimization theory, which posit that there is a single ideal life history that should exclude alternatives through natural/sexual selection (Schaffer, 1979; Stearns, 1980). More recently, researchers have adopted a game theoretic view of mating systems in which individual fitness varies based on the activities of others (Maynard Smith, 1982; Oliveira, Taborsky, & Brockmann, 2008; Shuster & Wade, 2003). In this framework, multiple genetically based mating tactics can persist in a population when the fitness of each is constrained by frequency‐dependent selection (FDS) (Gross, 1984; Shuster & Wade, 2003). By reducing the per‐capita reproductive success of a phenotype as it becomes more common, FDS should produce a stable distribution (or stable oscillations, Sinervo & Lively, 1996) of tactics in which alternatives experience equal average fitness (Shuster & Wade, 1991, 2003; but see Tomkins & Hazel, 2007). While the theoretical bases for the stability of ARTs are well‐developed (reviewed in Shuster & Wade, 2003; Taborsky et al., 2008) and supported empirically (Berejikian et al., 2010; Bleay, Comendant, & Sinervo, 2007), resolving why certain populations deviate from equilibrium conditions is important for understanding mating system evolution, and the potential consequences of fishery exploitation.

In anadromous Pacific salmon (*Oncorhynchus* spp.), most males compete aggressively on the spawning grounds for mating opportunities after reaching a large body size at sea. In addition to these typical “hooknose” males, males of certain species may also mature as “jacks,” which migrate to the ocean but return to freshwater to spawn earlier than the youngest females in the population (Quinn, 2005). Substantially smaller, and with more subtle secondary sexual characteristics than their older counterparts, jacks are generally ineffective at acquiring mates through intrasexual contests and female courtship (Gross, 1984). However, jacks possess numerous morphological and physiological specializations for achieving fertilizations through “sneaking,” including a higher gonadosomatic index (Flannery, Butts, Słowińska, Ciereszko, & Pitcher, 2013), improved sperm performance (Young, Conti, & Dean, 2013), and cryptic body coloration compared to older males (Gross, 1984). While either behavior is possible in both male phenotypes (Allen, Rich, & Quinn, 2007; Berejikian, Tezak, & LaRae, 2000), jacks and hooknoses achieve the majority of their matings through sneaking and fighting, respectively (Gross, 1984). In order for a juvenile salmon to become a jack, it must reach a critical growth or lipid accumulation threshold by a certain point in its life cycle (Shearer & Swanson, 2000; Thorpe, Mangel, Metcalfe, & Huntingford, 1998). As such, environmental (Koseki & Fleming, 2007; Vøllestad, Peterson, & Quinn, 2004) and genetic (Berejikian, Van Doornik, & Atkins, 2011; but see Hankin, Nicholas, & Downey, 1993; Barson et al., 2015) factors influential to juvenile growth and body condition can contribute to the developmental decision to mature as a jack. Importantly, there are also heritable differences among individuals in the threshold for early maturation (Aubin‐Horth, Bourque, Daigle, Hedger, & Dodson, 2006; Baum, Laughton, Armstrong, & Metcalfe, 2004; Piche, Hutchings, & Blanchard, 2008), such that alternative male phenotypes in salmon likely reflect underlying genetic variation in this trait as well (Hankin et al., 1993).

Evidence from theoretical and experimental studies suggests that alternative life histories of male salmon are maintained through FDS and can experience equal average fitness (Berejikian et al., 2010; Gross, 1984, 1985, 1991; Hutchings & Myers, 1994). In most populations, the percentage of males that mature as jacks remains relatively small over time (DeFilippo et al., 2018), presumably due to the stabilizing effects of FDS. However, some studies have questioned the feasibility of a stable evolutionary equilibrium between alternative male phenotypes in salmon, noting that extrinsic stochastic controls on juvenile growth and survival could preclude such an equilibrium (Koseki & Fleming, 2006, 2007). Moreover, precocious males can become quite prominent in some stocks, degrading fishery performance and posing an intractable challenge to management (Larsen et al., 2004; Zimmerman, Wes Stonecypher, & Hayes, 2003). As such, it is important to understand what regulates the realized dynamics of alternative life histories in salmon to assess how fisheries management decisions may impact their stability.

In a population of sockeye salmon (*O*. *nerka,* Walbaum, 1792) spawning in Frazer Lake on Kodiak Island, Alaska, jacks have recently been observed at extraordinarily high levels, accounting for over half of spawning males in some years (Foster, 2008). Because they are of little commercial value due to their small body sizes and perceived unmarketability, the elevated prevalence of jacks in Frazer Lake sockeye runs has led to substantial economic losses for local fisheries, and growing uncertainty about the population's future (Jackson & Keyse, 2015). In 2014 and 2015, mounting public concern over the issue led to a cull on jacks by management in an attempt to reduce their levels in the population. The financial and logistical burdens imposed by these efforts, coupled with concerns about potential negative effects on stock productivity and genetic diversity led to a decision in 2016 to discontinue culling (Foster & Schaberg, 2016). Here, we examine the temporal dynamics of jacks in Frazer Lake sockeye salmon to inform future management approaches to mitigating their proliferation. While previous experiments have identified proximate determinants of reproductive life history at the individual level, our approach integrates long‐term demographic and environmental data to explore what regulates a population's realized jack prevalence over time. Our results emphasize the importance of genetic factors in expression of the jack life history and reveal a novel mechanism arising through stochastic population dynamics that may ultimately determine the stability of ARTs in salmon.

## 2 METHODS

### 2.1 Study site

**Figure 1:**
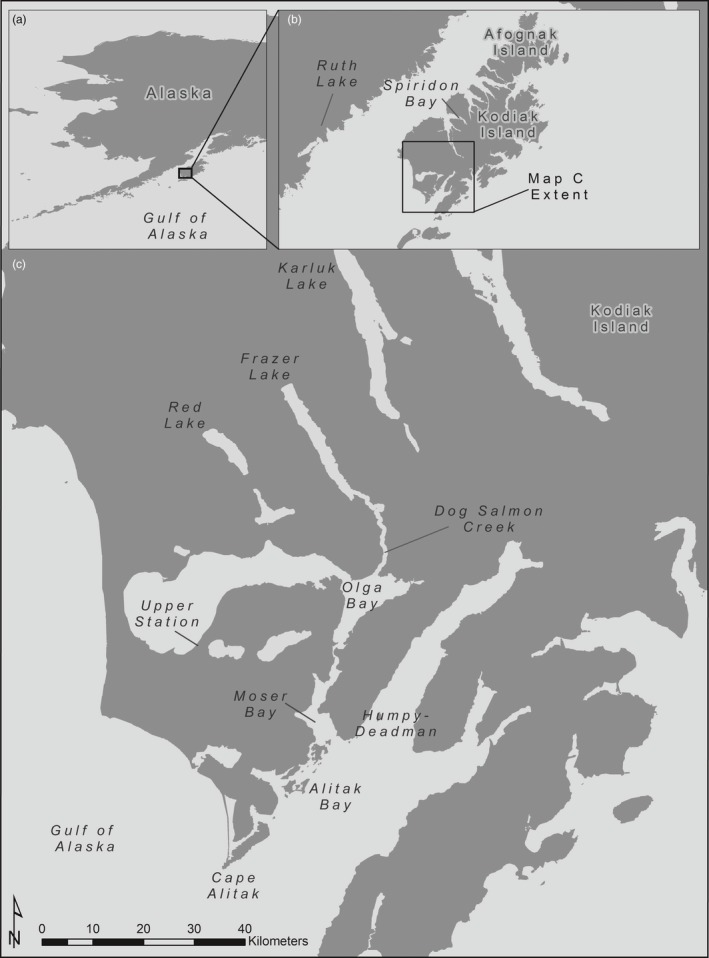
Map of Kodiak Island, with Frazer Lake and its associated donor lakes, harvest areas, and other relevant locations indicated

Located on the southern end of Kodiak Island, Alaska (57°15′N, 154°10′W), Frazer Lake drains to the southwest into Olga Bay via Dog Salmon Creek (Figure 1). An impassable waterfall on the upper portion of Dog Salmon Creek originally prevented upstream migration by anadromous salmon to Frazer Lake (Ruhl, 2017). In an effort to create an economically viable salmon run, sockeye adults, eggs, and fry were stocked in Frazer Lake from 1951 to 1971 using broodstock primarily from Red Lake (Ayakulik stock), and also Karluk Lake (Figure 1c) and Ruth Lake/Becharof Lake on the Alaska Peninsula (Figure 1b) (Blackett, 1979; Burger, Scribner, Spearman, Swanton, & Campton, 2000). Adult salmon returning to spawn were initially backpacked over the falls to access the lake until the construction of a fish ladder in 1962, followed by the installation of a second parallel fishway in 1979 (Kyle, Koenings, & Barrett, 1988). Run sizes increased substantially from 1974 to 1985, leading to concomitant declines in zooplankton biomass and juvenile body condition (Kyle et al., 1988). In an attempt to mitigate these density‐dependent effects on juvenile growth, nitrogen and phosphorus fertilizers were added to Frazer Lake from 1988 to 1992, leading to significant increases in zooplankton biomass (Kyle, 1994). There have been no further efforts to enhance the habitat quality of Frazer Lake since 1992. Since the development of a self‐sustaining run in Frazer Lake, this population has become one of the most commercially valuable stocks in the region. Harvest of Frazer Lake fish occurs within the Alitak district, primarily in a gillnet fishery operating in the Alitak, Moser, and Olga Bay sections, with a lesser degree of purse seine harvest in the Cape Alitak and Humpy‐Deadman areas as well (Figure 1c; Foster, 2008).

### 2.2 Data collection

In exploited salmon stocks, the number of fish that successfully migrate past fisheries and arrive on the spawning grounds in a given year is referred to as the “escapement.” The individuals within the escapement comprise that year's reproductive population, or “spawners,” and their offspring that reach maturity are the “return” from that “brood year.” Because age‐at‐maturity is variable within most populations, offspring produced from a given brood year reach maturity and spawn across multiple different calendar or “return years.” A “run” refers to a group of fish that share the same return year, which includes individuals produced from multiple brood years. The return year age composition is useful for understanding the contributions of each age class to the harvest and breeding population, while the brood year age composition reflects the maturation schedules of individual cohorts. Data on run size and age composition are collected by return year, and the abundance of each age class is lagged by its total age to calculate numbers‐at‐age produced from a given brood year in a process known as “run reconstruction.” The Alaska Department of Fish and Game (ADF&G) maintains such data for Frazer Lake sockeye salmon with information on cohorts from as early as the 1968 brood year, although the raw abundance and age composition data used to develop the run reconstruction are not available prior to 1986, when more intensive population monitoring began (Supporting information Table S1).

Each year, the ADF&G counts and samples adult sockeye salmon returning to spawn in Frazer Lake (described in Ruhl, 2017). Fish are counted as they enter Dog Salmon Creek and again when they pass through the fish ladders, where they are also sexed, and the number of jacks (based on visual identification and length measurements, e.g., Carlson, Rich, & Quinn, 2004) is recorded. The annual age composition of the escapement is determined by visual examination of scales (Gilbert, 1913; Mosher, 1969) taken from samples of adult salmon as they pass through the fish ladders. Frazer Lake sockeye salmon typically spend 1–3 years rearing in the lake, followed by an additional 1–3 years in the ocean, with 2 years being the most common period of lacustrine and marine residency for this population. All fish spend their first year of life overwintering in the gravel, but we do not include this period in our assignment of “freshwater age.” In sockeye salmon, “hooknose males” typically mature at ocean age‐2 or ocean age‐3 while jacks are defined as males with a marine residency of 1 year and were identified as such from the scale samples (ocean age‐1 females are very rare (Quinn, 2005)). Some age classes were only marginally represented in the age distribution (ocean age‐4/freshwater age‐4) and were removed to reduce the number of estimated parameters in our models (see Methods: Analysis). Individuals with freshwater and marine residencies of 1–3 years were retained in our analyses, generating a total of nine unique age combinations (e.g., ocean age‐1, freshwater age‐1 etc.) that accounted for ~ 98% of individuals in the population.

The age composition of harvested fish is determined from scale samples taken from commercial landings within the Alitak district (described in Foster, 2008; Moore, 2014). Due to the mixed stock nature of these fisheries, Frazer Lake fish are harvested with sockeye salmon from the Upper Station (South Olga Lakes) population (Figure 1C). Historically, harvested fish were assigned to their population of origin using age‐specific scale pattern analysis (SPA, Marshall et al., 1987) until 2001, when the SPA project was discontinued. Since then, the abundance and age composition of harvested Frazer Lake and Upper Station fish have been determined by assuming equal exploitation rates between stocks and inferring the relative contribution of each population to the harvest using the ratio of their escapements. Ratios are calculated as 3‐day moving averages based on specified travel times between the harvest areas and weirs at which daily escapements for both populations are enumerated (Foster, 2008).

We obtained time series of several environmental variables to evaluate as covariates when modeling the dynamics of jacks in Frazer Lake sockeye salmon (see Methods: Analysis). Annual Kodiak air temperatures were obtained from the National Weather Service Forecast Office, Fairbanks AK, and annual values of the North Pacific Gyre Oscillation (NPGO, Di Lorenzo et al., 2008)—an index of basin‐scale oceanic conditions relevant to salmon productivity—were downloaded from the online repository for these data (http://www.o3d.org/npgo/npgo.php). The average seasonal biomass of the main zooplankton genera comprising the diets of juvenile Frazer Lake sockeye salmon (*Daphnia* and *Bosmina,* Kyle et al., 1988) was estimated each year based on the protocol described in Ruhl (2017). An estimate of zooplankton biomass was not available for the first year in which covariate effects were evaluated, so a value was imputed using the average of the following 2 years’ estimates.

### 2.3 Analysis

Our analysis had three goals: (1) determining if there have been any changes in the proportion of Frazer Lake sockeye salmon maturing as jacks within cohorts over time (by brood year), (2) evaluating which, if any, environmental and demographic variables influence the proportion of individuals maturing as jacks within cohorts, and (3) comparing patterns of variability in the dynamics of the population's main ocean age classes by brood versus return year. For goal 1, we were interested in estimating the general trend in jack proportions over the history of this population, so we fit a simple Bayesian random walk model to values of brood year jack proportions calculated from the ADF&G run reconstruction. While the reconstructed brood year age composition is available for most of the population's history (as far back as brood year 1968), it does not have information on the sampling effort (e.g., scale sample sizes) for these early cohorts that would be necessary to reliably distinguish process from observation error in a state‐space framework. To explore finer‐scale temporal dynamics in the age composition of the population (goals 2–3), we developed a series of age‐structured Bayesian state‐space models (e.g., Fleischman, Catalano, Clark, Bernard, & Chen, 2013; Staton, Catalano, & Fleischman, 2017) for the time period in which the raw abundance and scale age composition data were available (return years 1986–2015). The core process components of these models take two general forms; the first quantifies the effects of various environmental and demographic covariates on the production of jacks within cohorts by brood year (goal 2). The second form estimates temporal patterns in the abundance of the population's main ocean age classes by both brood and return year (goal 3). In each model, the observation component operates by return year, accounting for measurement error associated with abundance estimates and scale samples from the harvest and escapement.

Goal 1: The conspicuously high jack proportions that motivated this study have occurred in the return year age composition, where jacks have comprised almost half of total (male plus female) runs in some years. To detect potentially less obvious temporal patterns in the proportion of individuals maturing as jacks within cohorts, we fit a biased (i.e., containing a linear trend) random walk to the time series of logit jack proportions by brood year based on values from the ADF&G run reconstruction:

(1) $$\begin{array}{c} J_{y}=J_{y-1}+u+w_{y}\\ w_{y}∼N\left(0,\sigma_{\mathrm{error}}\right) \end{array}$$

where (*J*_*y*_) represents the predicted logit jack proportion in brood year *y*,* w*_*y*_ is a normally distributed error term, and *u* is a logit‐linear trend parameter. The standard deviation of the error terms σ_error_ was drawn from a half‐Cauchy prior distribution with a location of 0 and a scale of 2.5 (Anderson, Branch, Cooper, & Dulvy, 2017), and the linear trend term (*u*) was drawn from a normal prior distribution with a mean of 0 and a standard deviation of 10. This model form was also fitted to the time series of jack and nonjack log abundances by brood year (also generated from the ADF&G run reconstruction) to compare temporal patterns in jack prevalence to variation in the returns of older individuals.

Goal 2: To identify environmental and demographic factors influential to the proportion of individuals maturing as jacks within cohorts**,** logit jack proportions (*J*_*y*_) across brood years were modeled as a lag‐1 autoregressive (AR(1)) process of the form:

(2) $$\begin{array}{c} \begin{array}{cc} J_{y}=\mu_{J}+\phi \left(J_{y-1}-\mu_{J}\right)+b\cdot c_{y}+w_{y} & \\ w_{y}∼N\left(0,\sigma_{\mathrm{proc}}\right) \end{array} \end{array}$$

In this equation, *ϕ* describes the autocorrelation in the state, μ_*J*_ represents the mean of the time series, *c*_*y*_ is a vector of covariate values for brood year *y* that affect the state, ***b*** is a vector of coefficients that describe the effect of each covariate, and the *w*_*y*_ are normally distributed process errors. We chose an AR(1) model based on inspection of autocorrelation in the raw data, and the expectation that low‐frequency environmental variation influencing juvenile growth and body condition would manifest as lag‐1 autocorrelation in the time series. Covariates evaluated for their effects on jack prevalence in a given cohort included the logit observed jack proportion in the males that sired that cohort, the log observed abundance of females that spawned that cohort (a proxy for rearing density, Schindler, Rogers, Scheuerell, & Abrey, 2005), the annual NPGO index, annual Kodiak air temperatures, and the log biomass of cladoceran zooplankton in Frazer Lake. The jack proportion and abundance of females in the spawners were each based on annual weir censuses of returning fish (see Methods: Data Collection). The effect of NPGO was considered as a 2‐year moving average, shifted forward by 2 and 3 years to encompass the window in which most fish from a given brood year would migrate to the ocean. Kodiak air temperature and Frazer Lake zooplankton biomass were both specified as 2‐year moving averages, shifted forward by 1 and 2 years to capture the period in which most fish from a given cohort would rear in the lake. Alternative reasonable combinations of lags and moving average lengths did not qualitatively alter model outcomes (Supporting information Figure S1). All model coefficients (***b***) were drawn from (assumed) vague normal prior distributions with means of zero and standard deviations of 10, and all covariates were *Z*‐scored. Phi (*ϕ*) was drawn from a uniform prior distribution with limits −0.999 to 0.999, μ_*J*_ was drawn from a normal prior distribution with a mean of 0 and a standard deviation of 20, and the process error standard deviation (*σ*_proc_) was drawn from a half‐Cauchy prior distribution with a location of 0 and scale of 2.5 (Anderson et al., 2017).

The information available for estimating brood year jack proportions consists of the scale samples and count data collected each return year from the harvest and escapement (See Methods: Data Collection). As such, model predictions of abundance and age composition for each of these sample sources needed to be generated from eq. 2 to evaluate the appropriate likelihoods. To do so, first the abundance of jacks $\left(B_{y}^{J}\right)$ and nonjacks $\left(B_{y}^{H}\right)$ were calculated from the estimated logit jack proportions (*J*_*y*_), and the abundance of the total return (*B*_*y*_):

(3) $$\begin{array}{c} B_{y}^{J}=B_{y}\frac{e_{y}^{J}}{1+e_{y}^{J}}\\ B_{y}^{H}=B_{y}-B_{y}^{J} \end{array}$$

The return to brood year *y* (*B*_*y*_) was modeled as a function of the total spawner abundance in that year (*S*_*y*_) according to the Ricker spawner–recruit relationship (Ricker, 1954):

(4) $$\begin{array}{cc} \ln(B_{y}) & =\ln\left(S_{y}\right)+\ln\left(\alpha \right)-\beta S_{y}+w_{y}\\ & \;\;\;w_{y}∼N\left(0,\sigma_{R}\right) \end{array}$$

where α is the productivity parameter and *β* is the inverse capacity term of the Ricker function, and the *w*_*y*_ are independent, normally distributed process errors. The broad uniform distributions that serve as reference priors for the Ricker spawner–recruit parameters (Millar, 2002) can be problematic for posterior sampling, so we used weakly informative priors for these terms based on estimates from other Kodiak sockeye salmon populations (Polum, Evans, & Dann, 2014; Schaberg, Foster, Wattum, & McKinley, 2016). Log productivity (ln (*α*)) and inverse capacity (*β*) were drawn from normal prior distributions with means of 0 and standard deviations of 20 and 0.1, respectively, and *σ*_*R*_ was drawn from a half‐Cauchy prior distribution with a location of 0 and a scale of 2.5. While the AR(1) form of the Ricker is commonly used for Alaskan salmon, we found no evidence of autocorrelation in the state residuals for equation 4.

Numbers‐at‐age by brood year (*B*_*y*,*a*_) were developed by multiplying the abundances of jacks and nonjacks by their respective maturity schedules:

(5) $$B_{y,a\in A^{k}}=B_{y}^{k}p_{y,a}^{k}$$

Here, *k* identifies whether a certain quantity pertains to jacks (*k* = *J*) or nonjacks (*k* = *H*) and *a* represents unique combinations of freshwater and marine ages. For simplicity, *a* is indexed numerically (e.g., *a *=* *1 represents ocean age‐1, freshwater age‐1, *a *=* *2 represents ocean age‐1, freshwater age‐2 etc.). *A* ^*k*^ denotes the set of age classes possible for jacks (*A* ^*J*^ = 1:3, representing ocean age‐1, freshwater ages‐1:3) and nonjacks (*A* ^*H*^ = 4:9, representing ocean ages‐2–3, freshwater ages‐1:3). Brood year age composition proportions for jacks and nonjacks were drawn from separate Dirichlet prior distributions, implemented as vectors of independent gamma‐distributed variables (*λ*_*y*,*a*_) divided by their sums (Gelman et al., 2013).

(6) $$\begin{array}{cc} p_{y,a}^{k} & =\frac{\lambda_{y,a}}{\sum_{a=A_{\delta }^{k}}^{A_{\omega }^{k}}\lambda_{y,a}}\\ & \lambda_{y,a}∼\text{Gamma}\left(\alpha =\xi_{a},\beta =1\right) \end{array}$$

where *a* ∊ *A* ^*k*^, and $A_{\delta }^{k}$ and $A_{\omega }^{k}$ are the first and last elements of *A* ^*k*^ ($A_{\delta }^{J}=1$, $A_{\omega }^{J}=3$, and $A_{\delta }^{H}=4$, $A_{\omega }^{H}=9$). In this parameterization of the gamma distribution, the inverse scale (rate) term (*β*) is a scaling factor that affects neither the mean nor the variability of the distribution and was fixed at 1 following Fleischman et al. (2013). Values of *ξ*_*a*_ were themselves drawn from vague gamma prior distributions with *α* = 0.001 and *β* = 0.001.

Numbers‐at‐age by return year were constructed by specifying the abundance of fish of age class *a* in return year *t* as the number of fish of age class *a* originating from brood year *y* = *t*−*T*_*a*_, where *T*_*a*_ represents the total age (gravel year plus freshwater and ocean ages) of age class *a*.

(7) $$R_{t,a}=B_{y=t-T_{a},a}$$

These values were then partitioned into numbers‐at‐age in the harvest (*C*) and escapement (*E*) according to age‐specific annual exploitation rates (*u*_*t*,*a*_).

(8) $$\begin{array}{c} R_{t,a}^{C}=R_{t,a}u_{t,a}\\ R_{t,a}^{E}=R_{t,a}-R_{t,a}^{C} \end{array}$$

where *u*_*t*,*a*_ were drawn from vague beta prior distributions:

(9) $$u_{t,a}∼\text{Beta}\left(0.5,0.5\right)$$

Goal 3: Because salmon acquire the majority of their adult body mass at sea, duration of marine residency is a particularly informative descriptor of a population's age structure. To estimate the degree of variability and coherence in the dynamics of the Frazer Lake population's main ocean age classes, we modeled their abundances by brood versus return year as two separate multivariate processes of the form:

(10) $$\begin{array}{cc} x_{y} & =x_{y-1}+w_{y}\\ w_{y} & ∼\text{MVN}(0,Q_{\mathrm{brood}}) \end{array}$$

(11) $$\begin{array}{cc} x_{t} & =x_{t-1}+w_{t}\\ w_{t} & ∼\text{MVN}(0,Q_{\mathrm{return}}) \end{array}$$

where ***x***_*y*_ and ***x***_*t*_ represent state vectors of the log abundance of ocean age‐1, ocean age‐2, and ocean age‐3 fish in brood year *y* and return year *t*, respectively. Process errors (***w***_*y*_, ***w***_*t*_) were assumed to be multivariate normally distributed with a mean of 0 and a variance–covariance matrix ***Q***. To estimate the degree of temporal coherence in the abundance of different ocean age classes by brood versus return year, we assumed a variance–covariance matrix structure with equal variance, and a single covariance term (*γ*) as an estimated parameter (i.e., variance $\left(\sigma_{\mathrm{proc}}^{2}\right)$ on the diagonal of the matrix and a single covariance term (*γ*) on all other entries). To avoid parameter draws leading to nonpositive definite matrices, *γ* was specified as the product of the variance $\left(\sigma_{\mathrm{proc}}^{2}\right)$ and the correlation (*ρ*) of the ***Q*** matrix. The process error standard deviation (*σ*_proc_) was drawn from a half‐Cauchy prior distribution with a location of 0 and a scale of 2.5, and *ρ* was drawn from a uniform prior distribution with limits of −0.999 to 0.999. Alternative methods to quantify synchrony among age classes by brood versus return year using cross‐correlation yielded qualitatively similar results (Supporting information Figure S2).

For each ocean age, its abundance ($e^{x^{i}}$, where *i *= ocean age‐1, ocean age‐2 or ocean age‐3) was multiplied by its freshwater age composition proportions $\left(p_{a}^{i}\right)$ to calculate numbers‐at‐age. For the model represented by equation 10, this calculation generated numbers‐at‐age by brood year:

(12) $$B_{y,a\in A^{i}}=e^{x_{y}^{i}}p_{y,a}^{i}$$

For the model represented by equation 11, this calculation produced estimates of numbers‐at‐age by return year:

(13) $$R_{t,a\in A^{i}}=e^{x_{t}^{i}}p_{t,a}^{i}$$

In each equation, *A* ^*i*^ represents the set of specific age classes comprising a given ocean age (i.e., freshwater ages; *A* ^*i*=1^ = 1:3, representing ocean age‐1, freshwater ages‐1:3, *A* ^*i*=2^ = 4:6, representing ocean age‐2, freshwater ages‐1:3). Proportions‐at‐freshwater age for each marine age class were drawn from separate Dirichlet prior distributions, implemented as in equation 6. For the brood year model (equation 10), estimates of numbers‐at‐age by return year were calculated as in equation 7. In both models, numbers‐at‐age in the run were assigned to the harvest or escapement as in equation 8.

For all process models (goals 2, 3), in each return year (*t*), the likelihoods for the abundance of Frazer Lake sockeye salmon in the harvest and escapement were assumed to follow log‐normally distributed observation processes.

(14) $$N_{t}^{s}∼\text{Lognormal}\left(\mu_{s}=\ln\sum_{a=1}^{\omega }R_{t,a}^{s},\sigma_{s}\right)$$

where *ω* represents the total number of all age classes (*a*) included in the analysis, $ln\sum_{a=1}^{\omega }R_{t,a}^{s}$ are the model‐predicted estimates of the log abundance of fish in return year *t* within a given sample source *s*, where *s *= catch (*C*) or escapement (*E*), and $N_{t}^{s}$ are the observed counts. Simultaneous estimation of process and observation error variance led to unresolvable divergent transitions during sampling (Betancourt, 2016; Monnahan, Thorson, & Branch, 2017), so we fixed the log‐normal observation error terms for the catch (*σ*_*C*_) and escapement (*σ*_*E*_) at 0.5 and 0.1, respectively, following a generalized run reconstruction model recently developed by Cunningham et al. (2017) that was applied to sockeye salmon in Bristol Bay, Alaska. Alternative values of these parameters did not qualitatively alter model outcomes (Supporting information Figures S3–S5).

Numbers‐at‐age observed in the scale samples taken from the harvest (***z*** ^*C*^) and escapement (***z*** ^*E*^) were assumed to follow a multinomial error structure. Scales were not collected from the harvest in certain years due to small run sizes and limited fishing, so age composition likelihoods for a given sample source were only evaluated for the subset of years in which data were available (*V* ^*s*^).

(15) $$\begin{array}{cc} z_{t\in V^{s}}^{s} & ∼\text{Multinomial}(\varepsilon_{t}^{s},\theta_{t}^{s})\\ \theta_{t,a}^{s} & =\frac{R_{t,a}^{s}}{\sum_{a=1}^{\omega }R_{t,a}^{s}} \end{array}$$

where $\theta_{t}^{s}$ are vectors of model‐predicted annual age composition proportions calculated from the numbers‐at‐age estimates $\left(R_{t,a}^{s}\right)$ and $\varepsilon_{t}^{s}$ are the effective sample sizes from a given source. We fixed $\varepsilon_{t}^{s}$ to be the minimum between each year's actual sample size or 1,000 for both the harvest and escapement (Fournier, Hampton, & Sibert, 1998).

In all models, posterior sampling was achieved via Hamiltonian Monte Carlo (HMC) No U‐turn Sampling (NUTS) routines executed in Stan (Stan Development Team, 2015) and implemented in R (R Core Team, 2015) using the Rstan package (Guo et al., 2016). Sampling occurred over four chains of 30,000 iterations each, with the first half of samples discarded as a “warmup” and one out of every five subsequent samples saved to develop the posterior distribution. Convergence was assessed using the Gelman‐Rubin diagnostic (Gelman & Rubin, 1992) and effective number of samples, as well as trace‐plots and autocorrelation plots of HMC chains. Posterior sampling was monitored for divergent transitions and low Bayesian Fraction of Missing Information (BFMI), neither of which were indicated. For all models, goodness of fit was assessed by comparing predictive distributions to observed data (posterior predictive checks; Supporting information Figures S6–S8), parameter estimation was validated using simulated data (Supporting information Figures S9–S11), and both state (process) and model (observation) residuals were examined for nonstationarity and autocorrelation, neither of which were evident.

## 3 RESULTS

**Figure 2:**
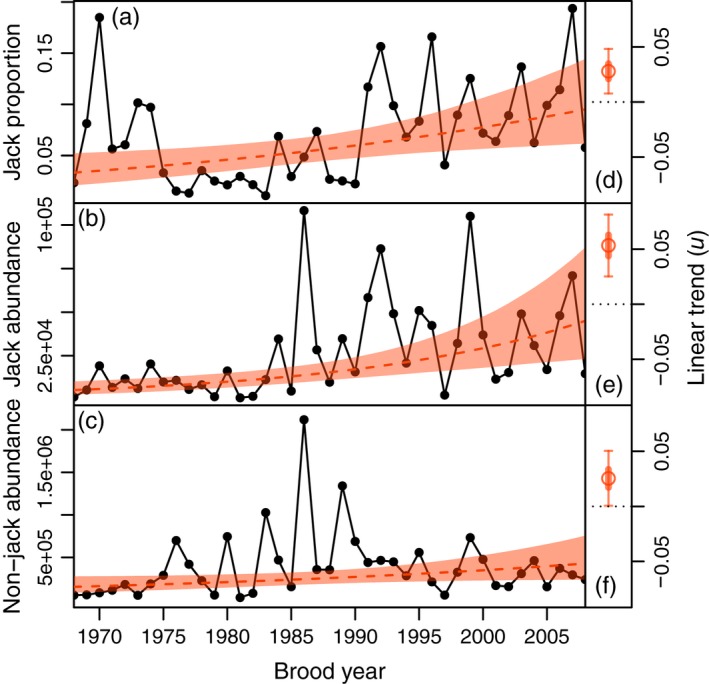
Time series of jack proportions (a), jack abundance (b), and nonjack abundance (c) within cohorts. Data (based on calculations from the ADF&G run reconstruction) are shown as black dots and lines, while model predictions from a biased random walk fitted to these data are shown in orange. Median posterior estimates are shown as dashed orange lines, with the 95% credible intervals depicted in transparent orange. The right‐hand panels depict the posterior distribution of the trend term (*u*) from model fits to brood year jack proportions (d), jack abundance (e), and nonjack abundance (f). Circles represent the median values of the posterior distributions, and thick and thin lines represent the 50% and 95% credible intervals, respectively

From brood years 1968 to 2008, the proportion of Frazer Lake sockeye salmon maturing as jacks exhibited a positive shift (Figure 2a). Jack proportions within cohorts (i.e., the age composition of returns from a given brood year) increased from an average of ~0.05 in the first half of this time period to ~0.09 in the latter half. The posterior median of the trend term (*u*, equation 1) in the biased random walk fitted to the logit of these data was 0.03 per year, with a 95% credible interval of 0.01 to 0.05 per year (Figure 2d). This increase in jack proportions reflects a positive trend in the abundance of jacks within the population as well (*u* median = 0.05, 95% credible interval = 0.03 to 0.08 log abundance per year) (Figure 2b, e). Interestingly, jack proportions were high from 1969 to 1974 (Figure 2a) when the Frazer Lake stock was still in the process of becoming established and total returns were relatively weak (Figure 2b, c). While there is evidence of a slight positive trend in nonjack returns from brood years 1968 to 2008 (*u*, median = 0.03, 95% credible interval 0.00 to 0.05 log abundance per year) (Figure 2c, f), nonjack abundance has exhibited a steady decline since the peak recruitment event of 1986 (Figure 2c). Model fits to this subset of data (not shown) indicated a median value for *u* of −0.05, with a 95% credible interval of −0.1 to −0.01 log abundance per year.

**Figure 3:**
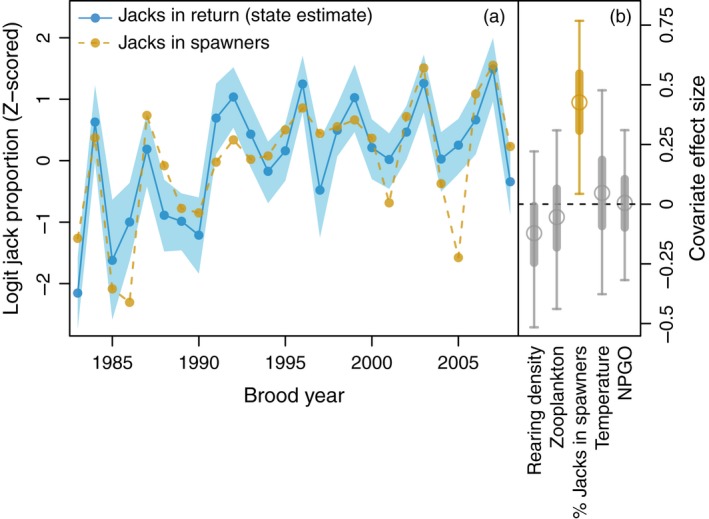
Covariate effects on jack prevalence within cohorts. Panel a depicts the model‐predicted estimates of jack proportions within the return from each brood year (blue). The median is represented by the solid line and points, and the 95% credible interval is indicated by the transparent boundary. Posterior distributions for the coefficients of all *Z*‐scored covariates that were evaluated are depicted in panel b, with the medians shown as circles, and the 50% and 95% credible intervals depicted as thick and thin lines, respectively. The posterior distribution for the covariate with the largest effect (proportion of spawning males that were jacks) is colored gold, and the time series for this variable is plotted alongside the state estimate to illustrate the relationship between these two variables. Note: jack proportions in the return are *Z*‐scored here to be on the same scale as the covariates, but in the fitted model they were not

**Figure 4:**
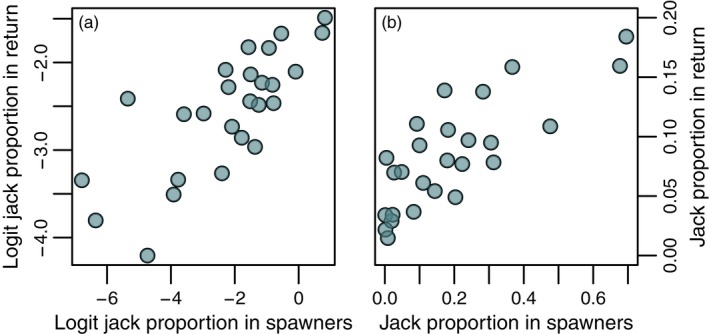
Spawner–return relationships for jack prevalence. The y‐ and x‐axes depict jack proportions in the return and the spawners, respectively, in logit (a) and linear (b) space

We found little evidence for any effect of environmental variation on the proportion of individuals maturing as jacks within cohorts. The coefficients for annual NPGO (median = 0.01, 95% credible interval = −0.32 to 0.31), air temperature (median = 0.05, 95% credible interval = −0.38 to 0.48), and log cladoceran zooplankton biomass in Frazer Lake (median = −0.05, 95% credible interval = −0.44 to 0.31) each suggested no consistent association with a cohort's jack prevalence. Similarly, there was limited support for a relationship between the log abundance of females in the breeding population (a proxy for fry production and rearing density (Schindler et al., 2005)) and the prevalence of jacks in the ensuing year‐class (median = −0.12, 95% credible interval = −0.52 to 0.22). However, the jack proportion in the return from a given brood year exhibited a strong, positive association with the prevalence of jacks among the males that sired that cohort (median = 0.43, 95% credible interval = 0.04 to 0.77) (Figures 3 and 4). The effect of this covariate was by far the largest among those considered (Figure 3b).

**Figure 5:**
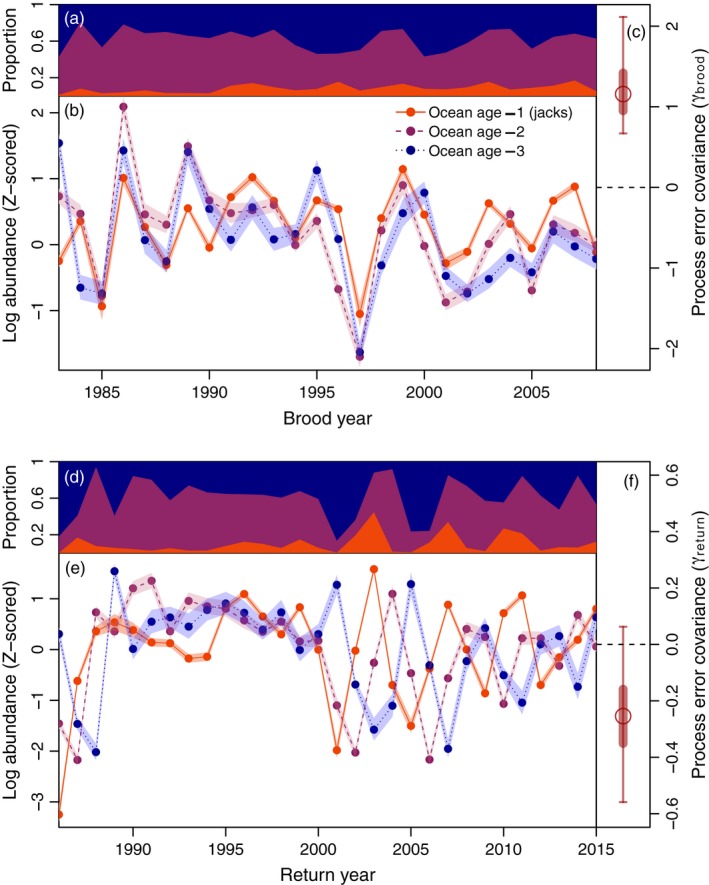
Temporal patterns in the marine age composition of the total (harvest plus escapement) population by brood and return year. Panels a and d show the posterior median estimates of the proportions of Frazer Lake sockeye salmon of ocean age‐1, 2, and 3 by brood year (a) and return year (d). Panels b and e depict *Z*‐scored state estimates of the log abundance of ocean age‐1, ocean age‐2, and ocean age‐3 fish by brood year (b) versus return year (e). Posterior medians are indicated by solid dots, while the 50% credible intervals are indicated as transparent boundaries. Panels c and f depict the posterior distribution of the process error covariance (*γ*) for each multivariate time series. The posterior median is indicated by a circle, while the 50% and 95% credible intervals are represented by thick and thin lines, respectively

Estimates of Frazer Lake sockeye salmon abundance by brood year indicated substantial interannual variation in cohort strength for this population (Figure 5b; median *σ*_*R*_ = 0.83, 95% credible interval = 0.64 to 1.12). Year‐to‐year variation in abundance was synchronous among marine age classes by brood year (*γ*_brood_, median = 1.16, 95% credible interval = 0.67 to 2.11), but distinctly asynchronous by return year (*γ*_return_, median = −0.25, 95% credible interval = −0.56 to 0.06) (Figure 5c, f). Return year dynamics appear uncorrelated throughout the time series, although the magnitude of this asynchrony increased conspicuously in 2003 (Figure 5e). This increase was initiated by two consecutive poor recruitments from the 1997 and 1998 brood years, followed by stronger recruitment from the 1999 brood year (Figure 5b). Most Frazer Lake sockeye salmon spend 2 years in the lake after overwintering in the gravel, such that the most common total ages for jacks, ocean age‐2, and ocean age‐3 fish are four, five, and six years respectively. Consequently, most ocean age‐2 and ocean age‐3 fish produced from the weak 1998 and 1997 brood years returned to spawn in 2003, together with jacks from the much stronger 1999 year‐class, resulting in approximately 45% of the 2003 run being composed of jacks. Similar, though less conspicuous “cohort mismatches” occurred in each return year characterized by high jack prevalence throughout the population's history (e.g., 1996, 1999, 2002, 2007, 2010, 2011, Figure 5b, d, e).

**Figure 6:**
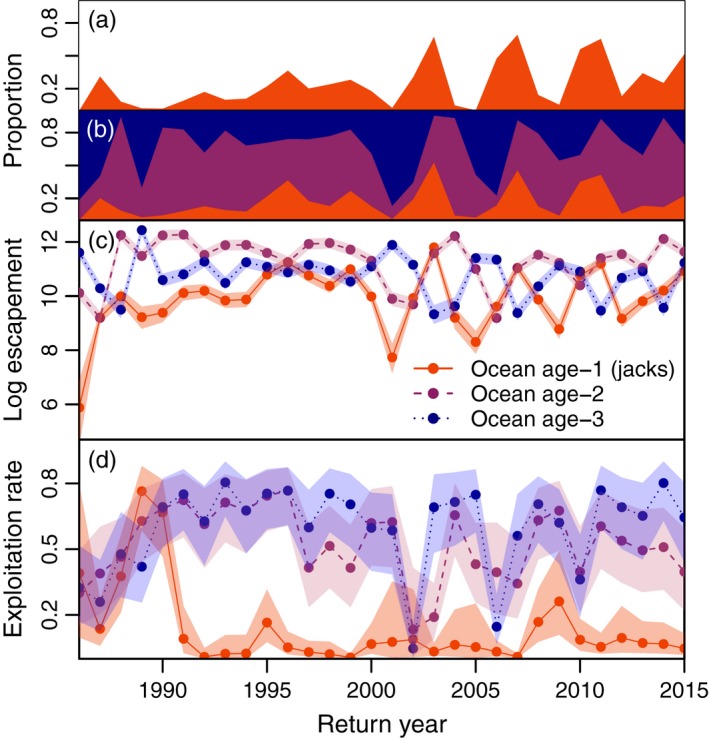
Estimated harvest rates and prevalence in the spawning escapement by ocean age. Panel a depicts the proportion of males in the escapement that were jacks (based on visual identification during annual weir censuses of returning fish). Panel b depicts median posterior estimates of the proportions‐at‐ocean age in the total spawning escapement (males plus females) by return year. Panel c shows the posterior estimates of the log abundance of each ocean age class in the spawning escapement by return year. Panel d depicts the estimated harvest rates for each ocean age class over time. For panels c and d, points and lines depict the median posterior estimates, and transparent boundaries indicate 95% credible intervals

From 1986 to 2015, median posterior estimates of annual exploitation rates for ocean age‐2 and ocean age‐3 fish averaged 0.52 and 0.60, respectively, while jacks were subject to average harvest rates of only 0.13 (Figure 6d). As a result, in years when jacks made up a large share of the total run, they usually comprised an even greater portion of the spawning escapement (Figure 6a–c). Moreover, while our harvest estimates were not stratified by sex, similar studies in other regions have identified greater fishery selectivity for (nonjack) male versus female sockeye salmon (Kendall & Quinn, 2013). For instance, in 2003, while jacks comprised roughly 45% of the total run, they accounted for 53% of the escapement and almost 70% of spawning males, even with a relatively low harvest rate (~ 0.22) on ocean age‐2 fish in that year (Figures 5d and 6a, b, d).

## 4 DISCUSSION

Our results indicate a distinct increase in the proportion of Frazer Lake sockeye salmon maturing as jacks between brood years 1968 and 2008. Temporal fluctuations in jack prevalence observed in other salmon populations have been attributed to environmental variation (Koseki & Fleming, 2007). The putative mechanism behind this association is that variation in factors influencing food availability, juvenile metabolism, or rearing density can affect the proportion of individuals reaching the growth/lipid accumulation threshold necessary for early maturation (Gross, 1991; Shearer & Swanson, 2000; Thorpe et al., 1998). Hatchery operations are thought to influence jacking rates as well, both through enhancing the growth and body condition of hatchery‐reared juveniles, and unintentional selection for younger age‐at‐maturity (Hankin, Fitzgibbons, & Chen, 2009; Unwin & Glova, 1997). However, we found little evidence for an association between the proportion of fish maturing as jacks and food availability, temperature, or variation in the marine environment relevant to salmon productivity, and limited support for an effect of rearing density. Moreover, the Frazer Lake stock is an entirely wild‐spawning (albeit introduced) population, and most hatchery propagation of sockeye salmon on Kodiak Island occurs near Spiridon Bay (Thomsen & Schrof, 2009), over 100 miles around the coast from Frazer Lake (Figure 1b). Instead, we found a strong positive relationship between the proportion of spawning males that were jacks and the proportion of individuals maturing as jacks in the ensuing cohort, suggesting that genetic factors are important in the determination of an individual's reproductive life history. This association is consistent with results from controlled breeding experiments that have demonstrated heritability (*h* ^*2*^ ~ 0.3–0.6) of the jack life history in other salmon species (Hankin et al., 1993; Heath, Devlin, Heath, & Iwama, 1994; Heath, Rankin, Bryden, Heath, & Shrimpton, 2002) as well as evidence from hatchery augmentation research (Unwin & Glova, 1997). While previous studies have indicated that the mating success of jacks is negatively frequency‐dependent (owing to greater competition for sneaking opportunities and refugia when jacks are common (Berejikian et al., 2010; Gross, 1984, 1985)), it is important to note that even if the average reproductive success of a phenotype declines at higher frequencies, the total share of fertilizations obtained by that tactic collectively can still increase as it becomes more prevalent (Ayala & Campbell, 1974). Our results suggest that this is likely the case for jacks in Frazer Lake sockeye salmon, at least over the range of values observed in this study (Figure 4).

Parental factors may strongly influence the proportion of individuals maturing as jacks in a given cohort, but this alone cannot explain why such proportions have increased over time. In fact, it is only when alternative tactics are genetically based that FDS is expected to stabilize their frequencies (Shuster & Wade, 2003; Taborsky et al., 2008). However, our analysis suggests that the volatile recruitment dynamics of Frazer Lake sockeye salmon may act to disrupt the stabilizing influence of FDS on this population's tactic frequencies. This is due to the fact that jacks and hooknose males differ in age‐at‐maturity, such that individuals of each phenotype co‐occurring on the spawning grounds are products of different cohorts. Because interannual variation in survivorship is synchronous within year‐classes, the ratio of jacks to hooknoses in the spawning population necessarily varies based on their relative cohort sizes. A similar pattern was described in Coho salmon (*O. kisutch,* Walbaum 1792) by Koseki and Fleming (2006), who noted that the difference in age‐at‐maturity between male phenotypes decoupled their synchronous fluctuations in abundance within cohorts. Our results show that when recruitment is highly variable, jacks from strong year‐classes may often spawn among older males from the much weaker recruitments of earlier brood years, leading to “cohort mismatches” in the breeding population that would not occur if recruitment were more stable. Consequently, jacks can repeatedly achieve substantial representation in the spawning population and subsequently high total fertilizations, increasing the prevalence of jacks in the next generation as well. These cohort mismatches are clearly responsible for the unusually high jack proportions observed in Frazer Lake sockeye runs by return year and are likely contributing to the increasing proportion of individuals maturing as jacks within year‐classes as well.

The variable population dynamics of Frazer Lake sockeye salmon may be caused or compounded by the unusual history of this population. Sockeye salmon were introduced into Frazer Lake less than seventy years ago using broodstock from neighboring populations (Ayakulik/Red Lake, Karluk Lake, Figure 1c), as well as Ruth Lake/Lake Becharof on the Alaska Peninsula (Figure 1b). Due to their philopatric life history, anadromous salmonids typically exhibit fine‐scale local adaptation to their natal habitats (Quinn, 2005). As the relatively recent descendants of individuals from populations that evolved in foreign environments, Frazer Lake fish may lack the same degree of local adaptation seen in other stocks, potentially leading to high variability in survivorship. We found synchronous patterns of abundance within year‐classes, indicating that survivorship is established in the freshwater or early marine phase of life, when individuals from a given cohort are exposed to common environmental conditions. It is primarily to the freshwater environment that local adaptation is thought to occur (but see Johnson & Schindler, 2013), such that incomplete adaptation to the Frazer Lake system may be contributing to the high variability in survivorship that this population exhibits. Indeed, noting substantial temporal variation in the spawning distribution, age composition, and abundance of Frazer Lake sockeye salmon, Burger et al. (2000) suggested that the population had not yet reached genetic or demographic equilibrium. As such, it is plausible that stochastic recruitment has not perturbed the Frazer Lake population's tactic frequencies away from an existing evolutionary equilibrium, but rather may have disrupted the development of any such equilibrium to begin with.

In addition to recruitment variability, there are other factors that have likely contributed to the increased prevalence of jacks in Frazer Lake sockeye salmon. Most notably, it is clear that fishery exploitation rates have been substantially lower for jacks than for ocean age‐2 or ocean age‐3 fish throughout most of the population's history. This is not unexpected, as Frazer‐bound fish are harvested primarily in a terminal gillnet fishery, which is strongly size‐selective (depending on the mesh sizes used; Foster, 2014). Indeed, analyses of the gillnet fishery for sockeye salmon in Bristol Bay, Alaska, showed disproportionate exploitation rates on older, larger individuals (Kendall & Quinn, 2009). Thus, in years when jacks from strong recruitments return to spawn among older males from weaker year‐classes, the numerical discrepancy in cohort sizes will be amplified by greater harvest of older individuals. Similarly, even in years when jacks are not a large portion of the total run, they may still comprise a substantial proportion of the spawning escapement due to size‐selective harvest. However, there are gillnet fisheries for sockeye salmon throughout Alaska, and the exploitation rates of Frazer Lake fish are similar to those in other regions (Kendall & Quinn, 2009). Yet, to our knowledge, increases in jack prevalence of the magnitude seen in Frazer Lake have not been documented in any other Alaskan sockeye salmon population (DeFilippo et al., 2018; ADF&G *unpublished data*). Furthermore, many years in which jacks comprised a large portion of the spawning escapement to Frazer Lake were also years with some of the lowest exploitation rates on older individuals (e.g., 2003, 2007, 2010; Figure 6). As such, it is unlikely that size‐selective harvest alone is responsible for the observed rise in jack prevalence, although it is likely a contributing factor.

While our analysis indicates that heritability of the jack life history, coupled with recruitment variation and size‐selective harvest, is responsible for the rising prevalence of jacks in Frazer Lake sockeye, there are alternative mechanisms that merit consideration. Our results suggest that enhanced jack prevalence in the breeding population arising from cohort mismatches leads to a high proportion of jacks in the ensuing cohort, which we attribute to the heritability of the jack life history. However, runs that exhibit cohort mismatches on the spawning grounds also tend to be relatively weak owing to reduced representation of older age classes that are usually numerically dominant. Therefore, cohorts resulting from these breeding events could be more likely to produce jacks due to reduced spawner abundance, fry production, and density‐dependent competition among juveniles. Such reduced density dependence could enhance juvenile growth and body condition within a cohort such that more individuals are likely to mature as jacks, creating a spurious association between the prevalence of jacks in the spawning population and its offspring. We included female spawning abundance as a covariate in our model to account for this possibility, the effect of which was much weaker than the prevalence of jacks in the breeding population. Nonetheless, the effect of rearing density was predominantly negative as expected, and stronger than any of the environmental variables considered. As such, it is possible that reduced density dependence may be contributing to the effects of cohort mismatches in the breeding population on the prevalence of jacks in the next generation, in conjunction with the heritability of the jack life history. However, while total run size fluctuates, the fixed escapement policy under which the Frazer Lake population is managed may dampen any density‐dependent effects on jack prevalence. In addition, it is worth noting that while the primary donor population to Frazer Lake (Ayakulik) has not shown any apparent shifts in jack prevalence, it does exhibit jack proportions that are relatively high compared to other Alaskan sockeye salmon stocks (Supporting information Figure S12; DeFilippo et al., 2018). As such, Frazer Lake fish may have been predisposed toward high jacking rates due to their ancestry, although this alone cannot explain the increased prevalence of jacks in recent decades.

The nutrient fertilization of Frazer Lake may also have contributed to the rise in this population's jack prevalence. Such an effect would presumably occur via an increase in ecosystem primary productivity, zooplankton biomass, and subsequently, juvenile growth and body condition. However, we found no evidence of a relationship between zooplankton biomass and jack prevalence among cohorts. While there were pronounced increases in jack proportions within the 1991 and 1992 year‐classes (Figure 2a), which were likely exposed to the effects of fertilization, closer examination of the data shows elevated jack proportions in the spawners that sired these cohorts (Figure 3a), owing to variable recruitment dynamics and size‐selective harvest (Supporting information Figure S13; Figure 6d). Moreover, the fertilization of Frazer Lake only occurred from 1988 to 1992, and it is unlikely that this brief episode could be responsible for the rising prevalence of jacks in recent years. Nutrient fertilization campaigns of sockeye salmon rearing lakes were historically numerous, and the results of these efforts have been described in great detail (Barraclough & Robinson, 1972; Budy, Luecke, & Wurtsbaugh, 1998; Hilborn & Winton, 1993; Hyatt, McQueen, Shortreed, & Rankin, 2004; Hyatt & Stockner, 1985; Kyle, 1994; Lebrasseur et al., 1978; Mazumder & Edmundson, 2002). However, despite such intensive study, to our knowledge there is no published account of a similar shift in jack prevalence following the nutrient fertilization of a sockeye salmon nursery lake.

It is not clear how the distribution of alternative male phenotypes in Frazer Lake sockeye salmon will behave in the future, but it seems unlikely that FDS could maintain a stable equilibrium in this stock so long as current levels of recruitment stochasticity persist. Importantly, substantial fluctuations in year‐class strength occur in many salmon populations (White, Botsford, Hastings, & Holland, 2014), suggesting that stable equilibria between alternative life histories may be difficult to achieve in other stocks as well. The heritable trait through which FDS operates in salmon is likely the growth or lipid accumulation threshold for precocious maturation (Gross, 1991; Shearer & Swanson, 2000). As such, the relative reproductive success of a tactic should also determine the proportion of offspring inheriting maturation thresholds that will dispose them toward expressing that tactic (Hankin et al., 1993). Repeated over generations, this process can produce an evolutionarily stable distribution of threshold genotypes that reflects the frequency‐dependent fitness functions of alternative tactics (Hutchings & Myers, 1994; Shuster & Wade, 2003). Importantly, this mechanism assumes that the ratio of male phenotypes on the spawning grounds reflects the current distribution of threshold values in the population. However, if the ratio of jacks to hooknoses in the breeding population each year depends more on their respective cohort sizes, as our results suggest, then the frequency‐dependent mating success of individuals will be effectively decoupled from their maturation decisions, causing the fitness payoffs associated with a particular threshold value to vary over time. As Koseki and Fleming (2006) noted, this dynamic should make an evolutionary equilibrium between male phenotypes more difficult to achieve. Our results support this assertion, suggesting that unless recruitment is constant over time, the ratio of male mating phenotypes in the breeding population will be repeatedly perturbed—each time affecting the distribution of threshold values, and ultimately tactic frequencies in the ensuing cohort (Hankin et al., 1993). Consequently, the realized frequencies of alternative male phenotypes in some populations may reflect the magnitude and pattern of these perturbations rather than the conditions of a stable equilibrium maintained by FDS, such that jacks would be more common in stocks with highly variable dynamics. While we were able to observe a discernable shift in tactic frequencies associated with recruitment variation in Frazer Lake sockeye salmon, this is likely a product of the unique opportunity presented by this stock to observe the development of a nascent population.

This study contributes to a growing body of work emphasizing the importance of evolutionary factors in the management of exploited populations (Allendorf & Hard, 2009; Heino, Díaz Pauli, & Dieckmann, 2015; Heino et al., 2013; Kendall, Dieckmann, Heino, Punt, & Quinn, 2014; Kuparinen & Hutchings, 2016; Kuparinen & Merilä, 2007). Most studies on harvest‐induced evolution have focused on continuous life history traits (e.g., size and age‐at‐maturity), while consideration of discontinuous variation (i.e., alternative maturation/reproductive phenotypes) is comparatively scarce, despite the large effects such variation can have on population age/size structure and fishery performance (Larsen et al., 2004; Myers, 1984; Zimmerman et al., 2003). Our findings suggest that genetic factors are important in the determination of an individual's mating phenotype, such that exploitation methods consistently targeting larger, older salmon may lead to selection in favor of jacks. Moreover, our study indicates that recruitment variation can lead to cohort mismatches in the breeding population that override the stabilizing effects of FDS and increase the total fertilizations obtained by jacks beyond what would be expected under equilibrium conditions. As such, managers seeking to control the prevalence of jacks in highly variable populations may need to develop methods to limit their presence on the spawning grounds. Increasing exploitation rates on jacks by using harvest methods that are less size‐selective may be one way to achieve this. Alternatively, the development of passive mechanisms to exclude some jacks from the spawning grounds that are not as financially or logistically demanding as culling may also be viable. Stabilizing population dynamics is likely to be the most effective management solution (avoiding both the high jack proportions in the run, and in the ensuing cohort), but also the least realistic to implement. Indeed, understanding the causes of variation in fish populations is a fundamental goal of fisheries science (Sissenwine, 1984; Smith, 1994), and it may be safely assumed that stocks would be managed to minimize variability if scientists knew how to accomplish this. Nonetheless, biologists have identified several anthropogenic factors that can amplify population variability, such as high exploitation rates (Essington et al., 2015; Hsieh et al., 2006; Shelton & Mangel, 2011), and reduced biocomplexity (Carlson & Satterthwaite, 2011; Griffiths et al., 2014; Moore, Mcclure, Rogers, & Schindler, 2010). Managing stocks with these factors in mind may be useful in minimizing variability and avoiding the undesirable proliferation of jacks. However, sources of variation differ between populations (Szuwalski, Vert‐Pre, Punt, Branch, & Hilborn, 2015; Vert‐pre, Amoroso, Jensen, & Hilborn, 2013) and identifying the drivers of demographic variability specific to a given stock will likely be necessary to develop effective management approaches for stabilizing its dynamics.

## DATA ARCHIVING STATEMENT

The historical numbers‐at‐age (brood table) data and covariate values used in this study are archived on Figshare under “DeFilippo et al., 2018 Evolutionary Applications Data,” https://doi.org/10.6084/m9.figshare.7040075
