# Supplementary material for: Recruitment variation disrupts the stability of alternative life histories in an exploited salmon population
Source: Evol Appl. 2018 Dec 1;12(2):214–29. doi: 10.1111/eva.12709 (PMC6346651; doi:10.1111/eva.12709)
Supplement: Supplementary file 1 [file EVA-12-214-s001.pdf]

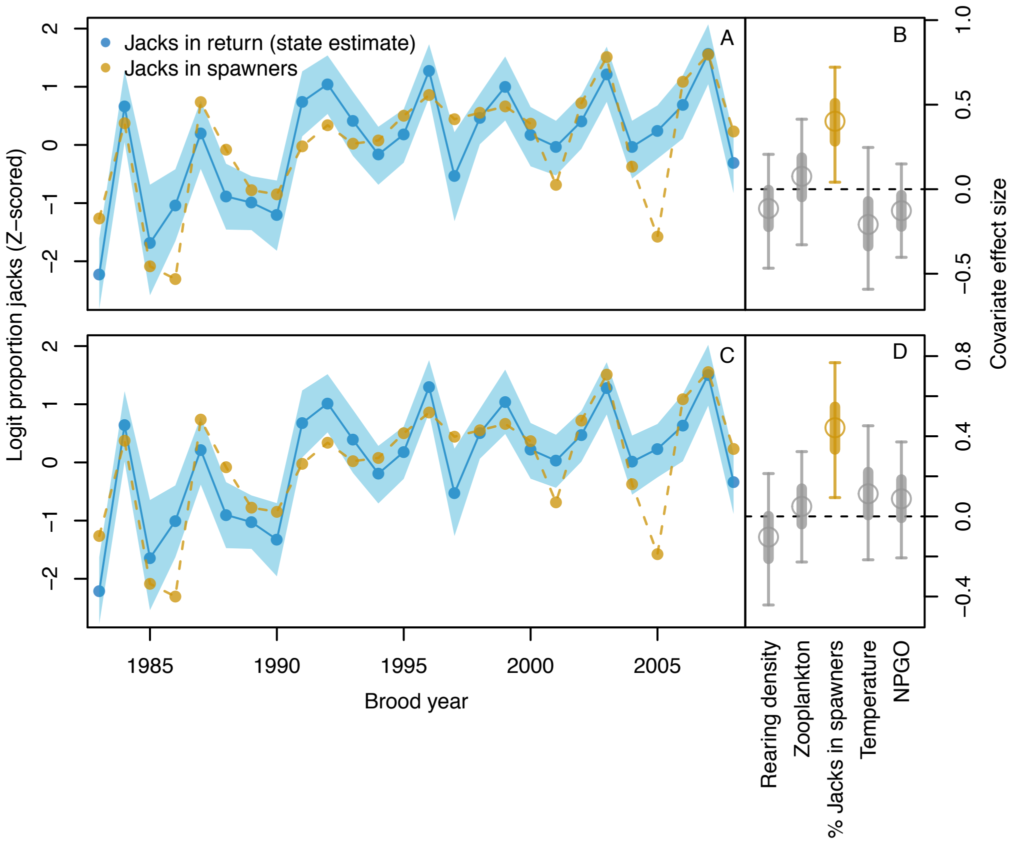

Figure S1. Sensitivity of state estimates and covariate effect sizes to alternative lags and moving average windows. In panels A-B, temperature is specified as a moving average of the current year and the next year to capture potential effects of temperature on developing eggs and fry, NPGO is shifted forwards by two years, and zooplankton biomass is set as a 2-year moving average, shifted forwards by 1 and 2 years (as in the main figure). In panels C-D, temperature and zooplankton biomass are both shifted forwards by 2 years to capture their effects towards the end of freshwater residency, and NPGO is shifted forward by 3 years.

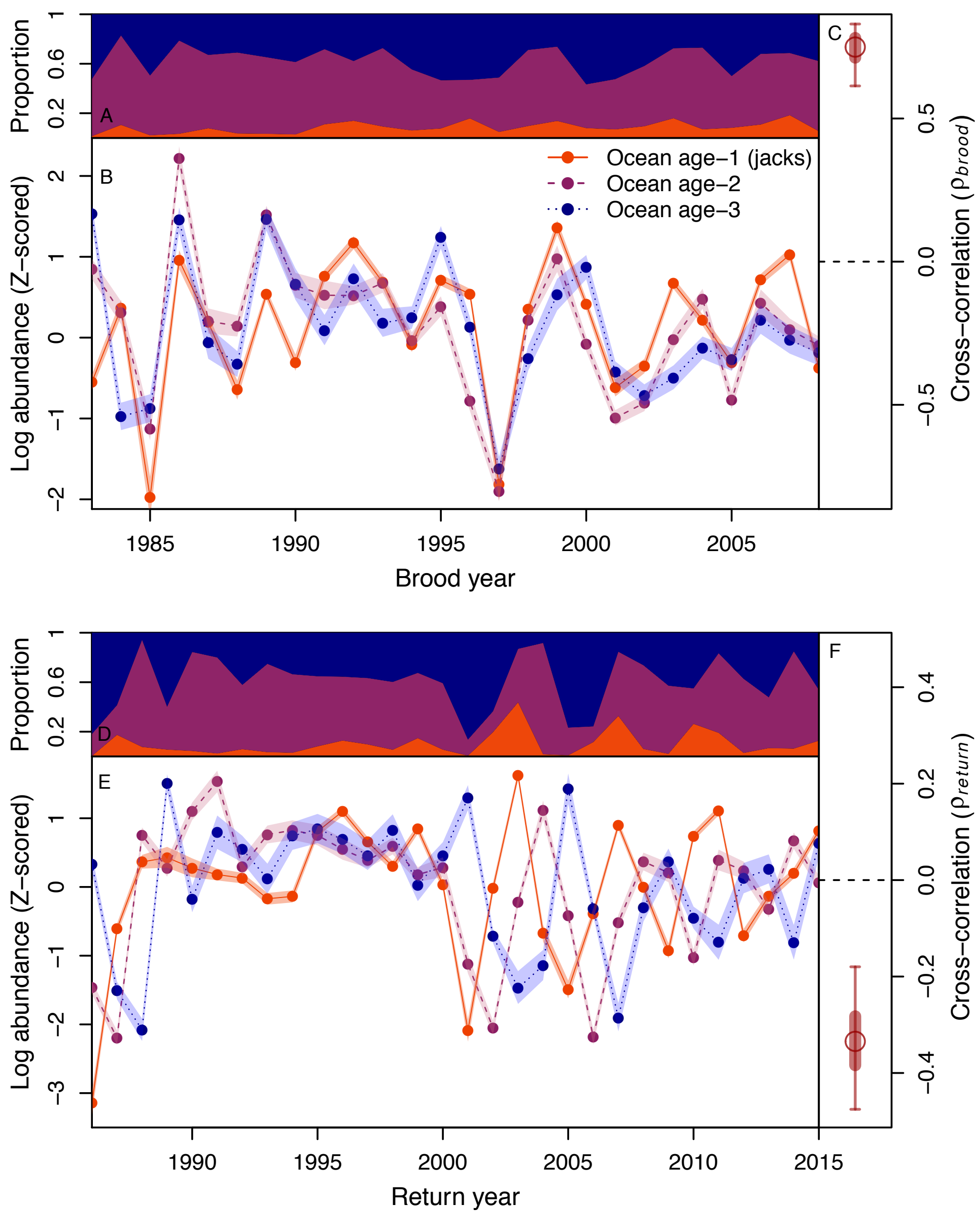

Fig S2. Results of figure 5 based on output generated solely from the model represented by eq. 2. (goal 1) As in figure 5 in the main text, panels A and D show the posterior median estimates of the proportions of ocean age-1, 2, and 3 fish by brood year (A) and return year (D). Panels B and E depict Z-scored state estimates of the log abundance of ocean age-1, 2, and 3 fish by brood year (B) versus return year (E). Posterior medians are indicated by solid dots, while the 50% credible intervals are indicated as transparent boundaries. Panels C and F depict the posterior distribution of the cross-correlation coefficient between jacks (ocean age-1), and the hooknose age classes (ocean age-2 plus ocean age-3) by brood year (ρ_{brood}) and return year (ρ_{return}). The posterior median is indicated by a circle, while the 50% and 95% credible intervals are represented by thick and thin lines respectively.

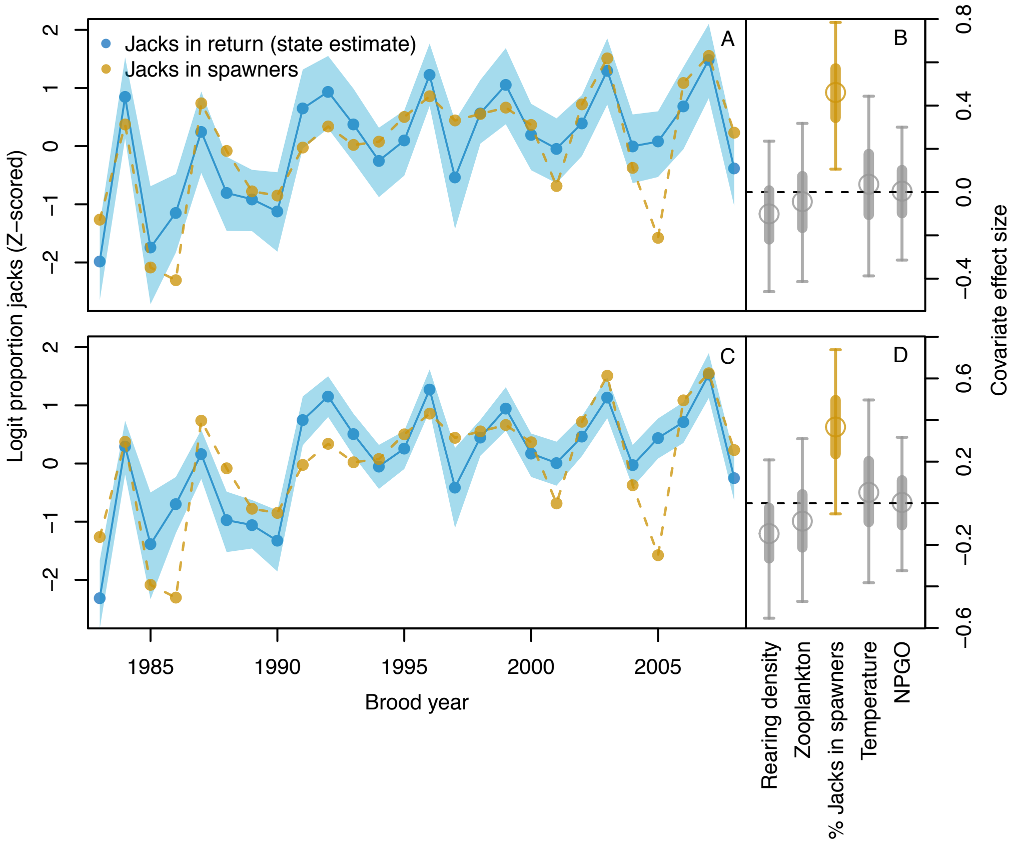

Figure S3. Sensitivity of state estimates and covariate effect sizes to alternative values of observation error variance for the harvest and escapement counts. Panels A and B represent the results of Figure 3 with higher observation error ($\sigma_C = 0.75$, $\sigma_E = 0.15$), while panels C and D represent the results under a lower observation error scenario ($\sigma_C = 0.25$, $\sigma_E = 0.05$).

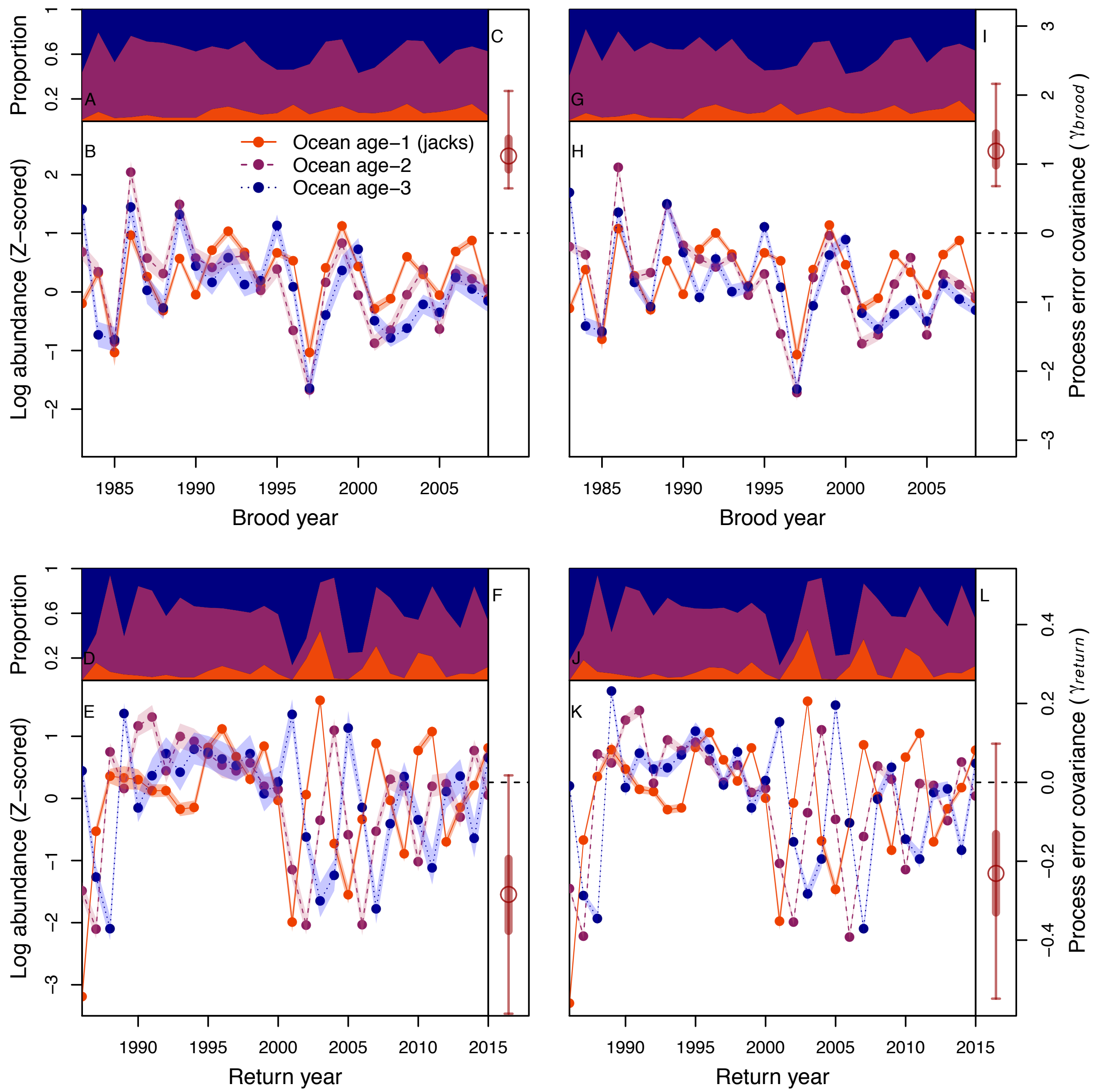

Figure S4. Sensitivity of state and covariance estimates to alternative values of observation error variance for the harvest and escapement counts. Panels A through F represent the results of figure 5 with higher observation error (σ_C = 0.75, σ_E = 0.15), while panels G through L represent the results under a lower observation error scenario (σ_C = 0.25, σ_E = 0.05).

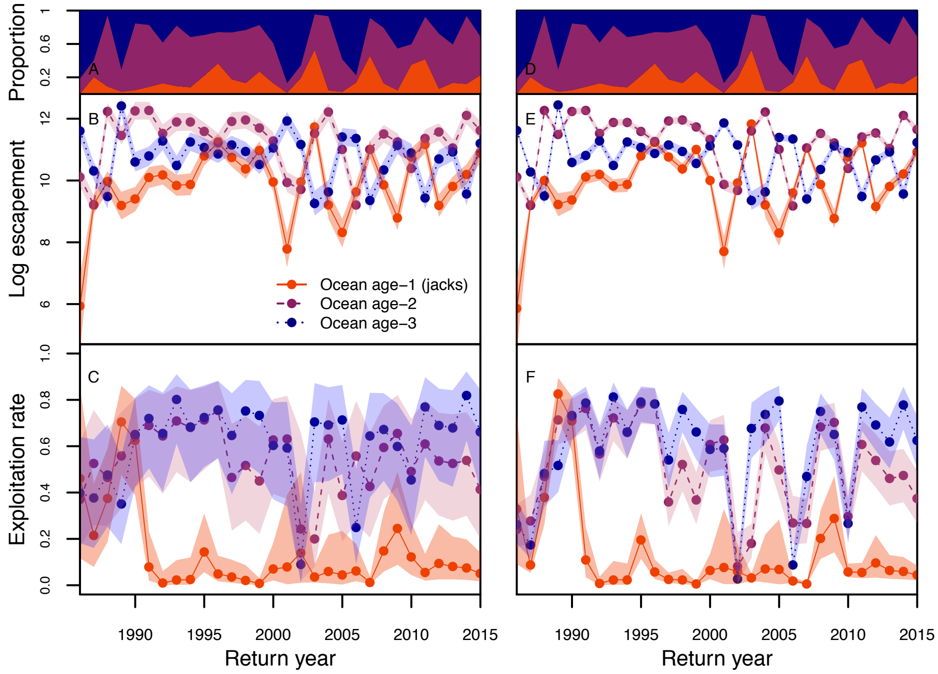

Figure S5. Sensitivity of harvest and escapement estimates to alternative values of observation error variance for harvest and escapement counts. Panels A through C represent the results of Figure 6 with higher observation error ($\sigma_C = 0.75$, $\sigma_E = 0.15$), while panels D through F represent the results under a lower observation error scenario ($\sigma_C = 0.25$, $\sigma_E = 0.05$).

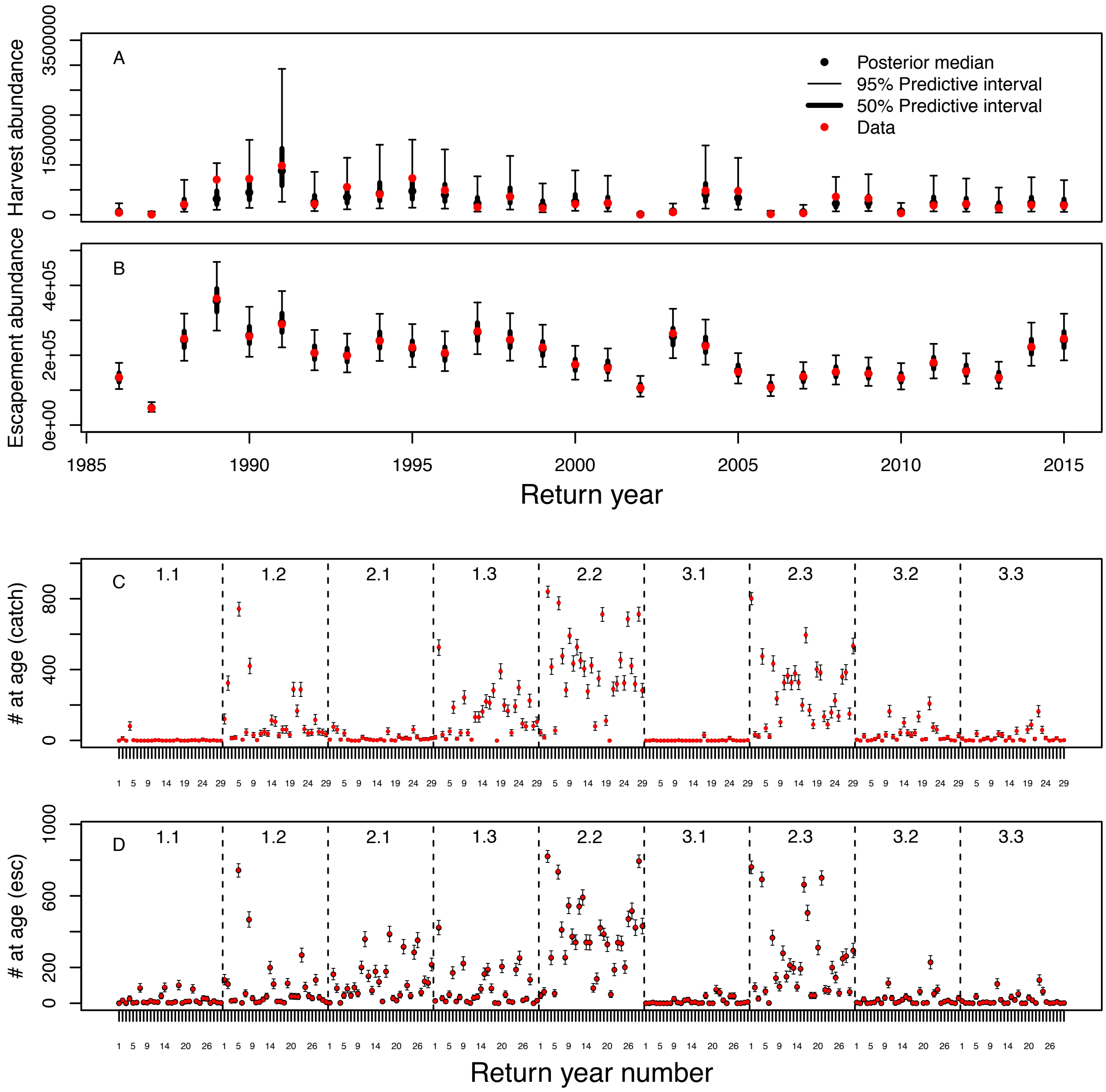

Figure S6. Posterior predictive check for the univariate model of brood year jack proportions (eq. 2). Panels A and B depict the predictive distributions relative to the observed data for the harvest and escapement abundance respectively. Panels C and D depict the predictive distributions relative to the observed data for the age composition of the harvest and escapement respectively. The predictive distributions for each age group across all years are separated by dotted lines. The labels represent freshwater and marine age separated by a decimal.

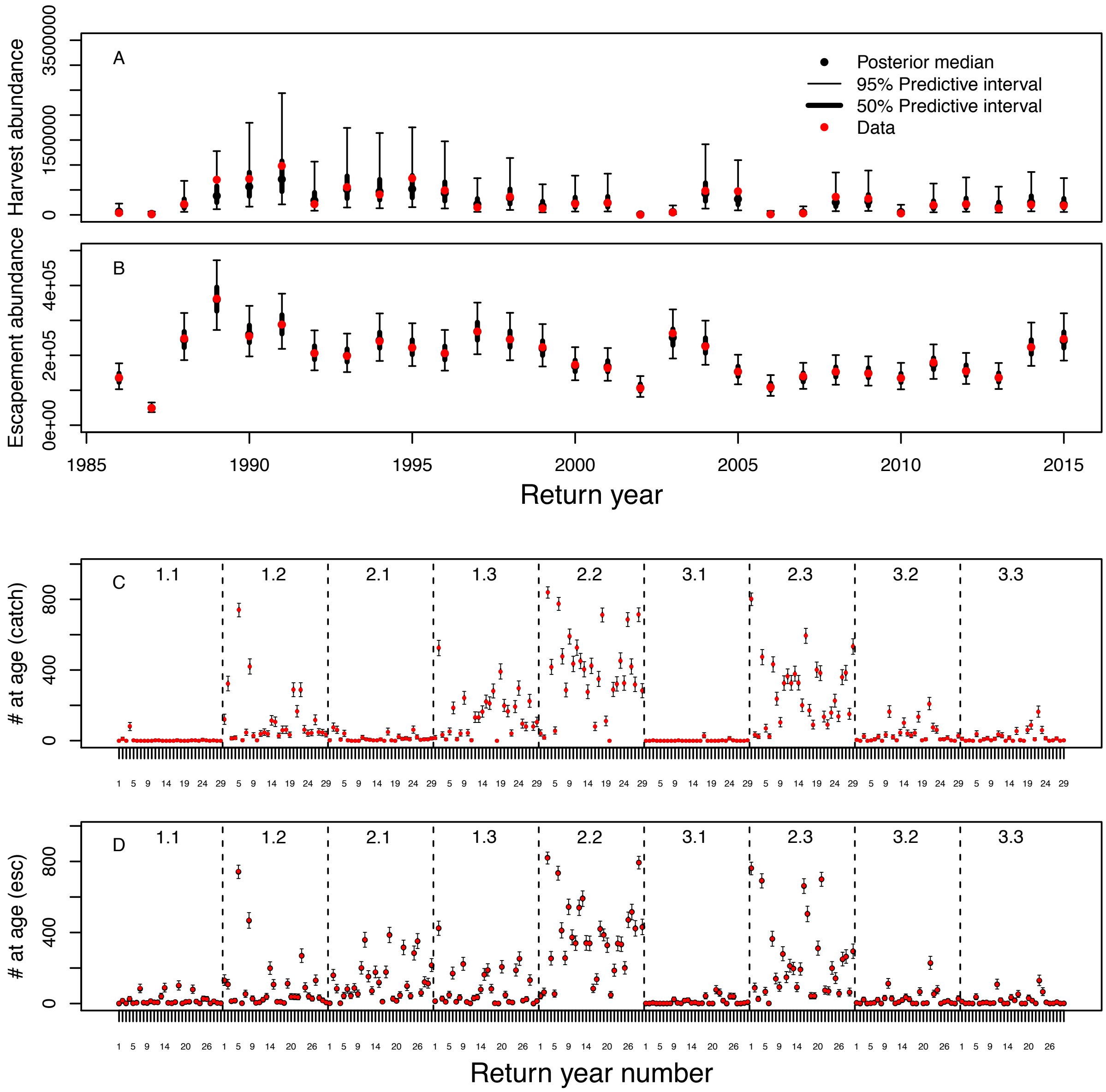

Figure S7. Posterior predictive check for the multivariate model of return year abundance of ocean age classes (eq. 11). Panels A and B depict the predictive distributions relative to the observed data for the harvest and escapement abundance respectively. Panels C and D depict the predictive distributions relative to the observed data for the age composition of the harvest and escapement respectively. The predictive distributions for each age group across all years are separated by dotted lines. Labels represent freshwater and marine age separated by a decimal.

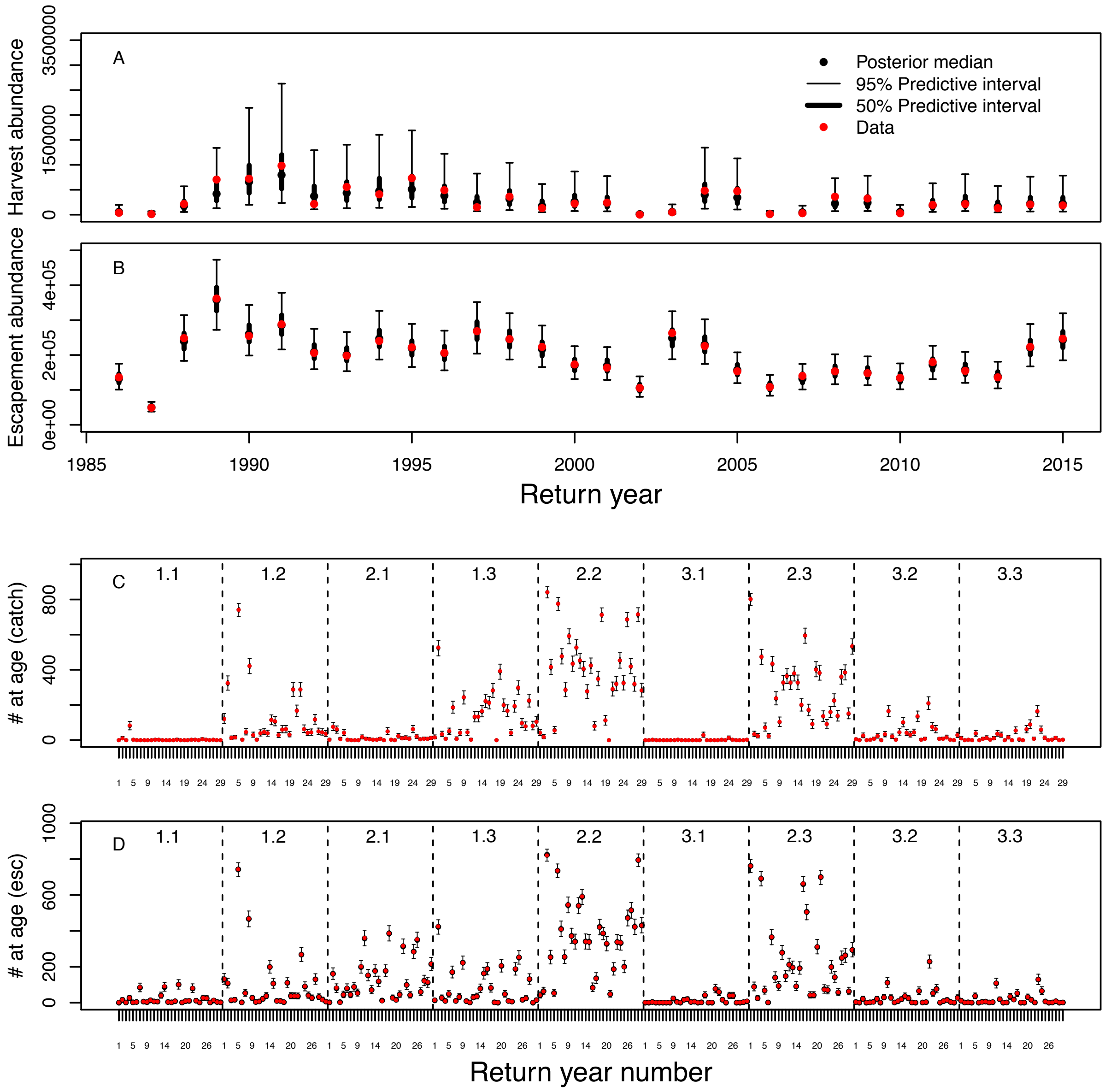

Figure S8. Posterior predictive check for the multivariate model of brood year abundance of ocean age classes (eq. 10). Panels A and B depict the predictive distributions relative to the observed data for the harvest and escapement abundance respectively. Panels C and D depict the predictive distributions relative to the observed data for the age composition of the harvest and escapement respectively. The predictive distributions for each age group across all years are separated by dotted lines. Labels represent freshwater and marine age separated by a decimal.

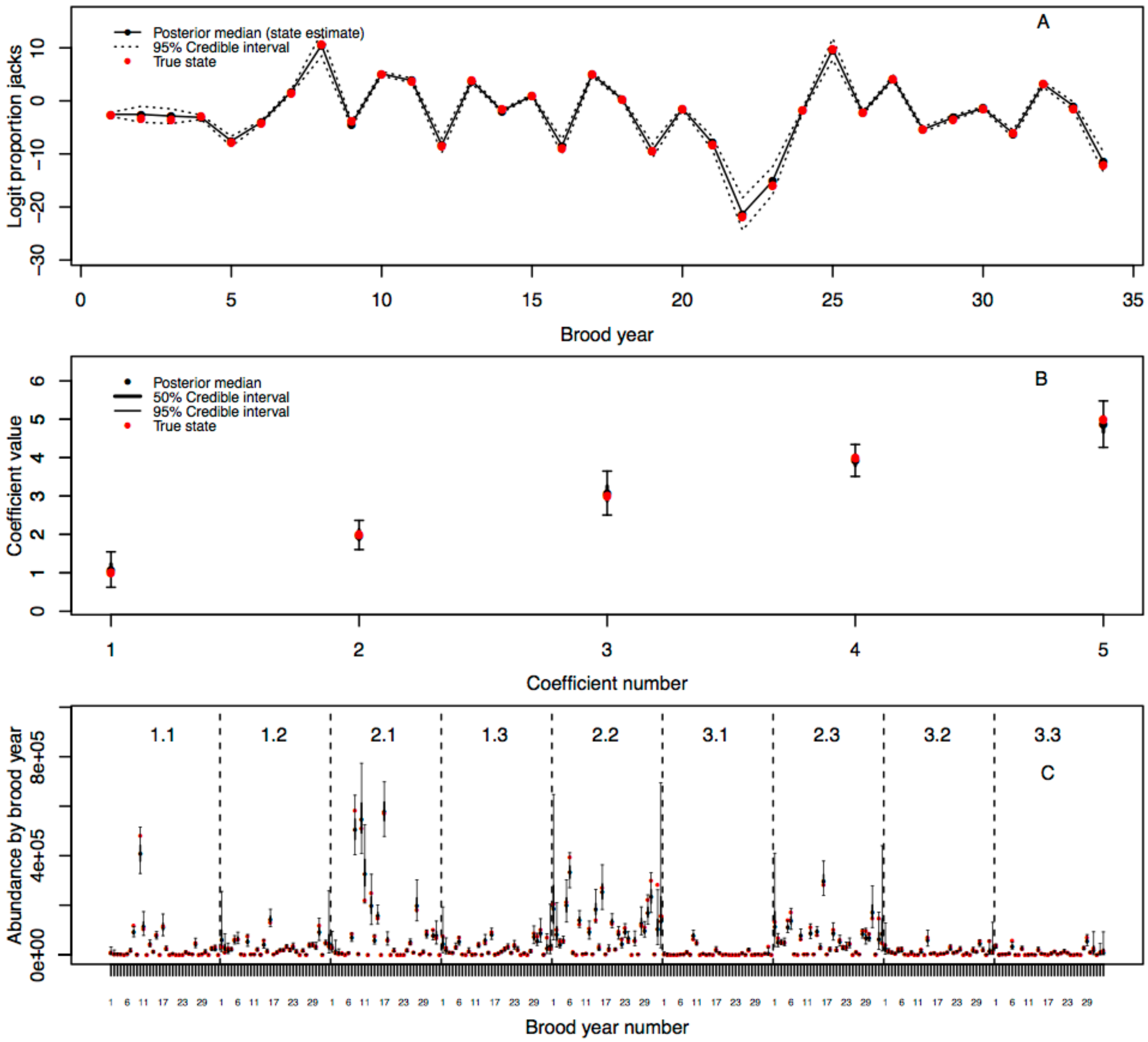

Posterior median (state estimate)
95% Credible interval
True state
A
Logit proportion jacks
Brood year
Posterior median
50% Credible interval
95% Credible interval
True state
B
Coefficient value
Coefficient number
1.1
1.2
2.1
1.3
2.2
3.1
2.3
3.2
3.3
C
Abundance by brood year
Brood year number

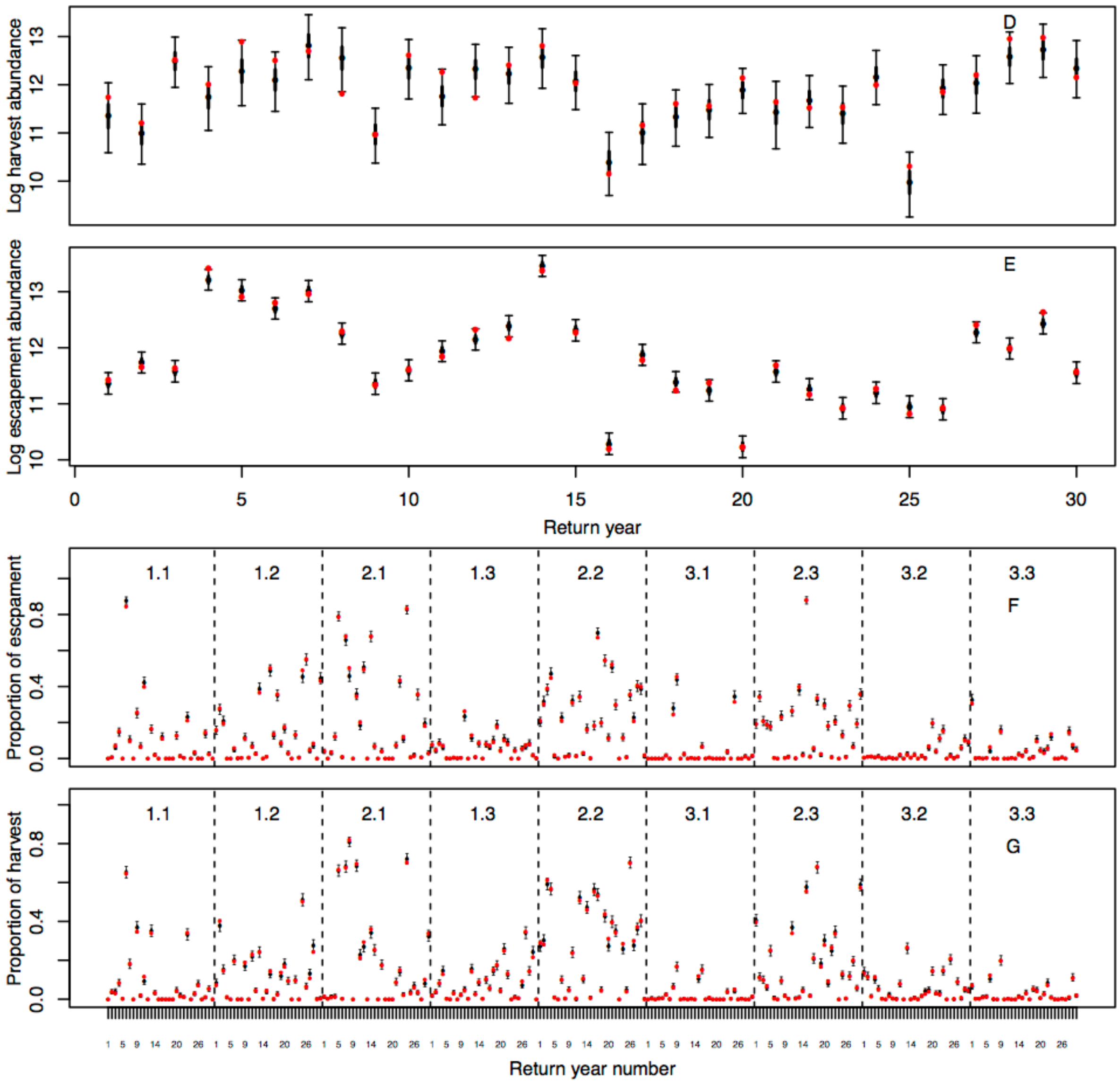

Figure S9. Model fit to simulated data for the univariate model of brood year jack proportions (eq. 2). Panel A depicts the credible intervals for estimated jack proportions relative to the true state values specified in the simulated data. Panel B depicts estimates for the coefficients of 5 covariates relative to the true values specified in the simulated data. Panel C depicts the credible intervals for estimated numbers-at age-by brood year relative to the values specified in the simulated data. Panels D and E depict the credible intervals for the model-predicted estimates of log abundance ($\ln \sum_{a=1}^{\omega} R_{t,a}^{s}$) relative to the values specified in the simulation for the harvest and escapement respectively. Panels F and G depict the credible intervals for the model-predicted estimates of age composition proportions ($\boldsymbol{\theta}_t^s$) over time relative to the values specified in the simulation for the escapement and harvest respectively.

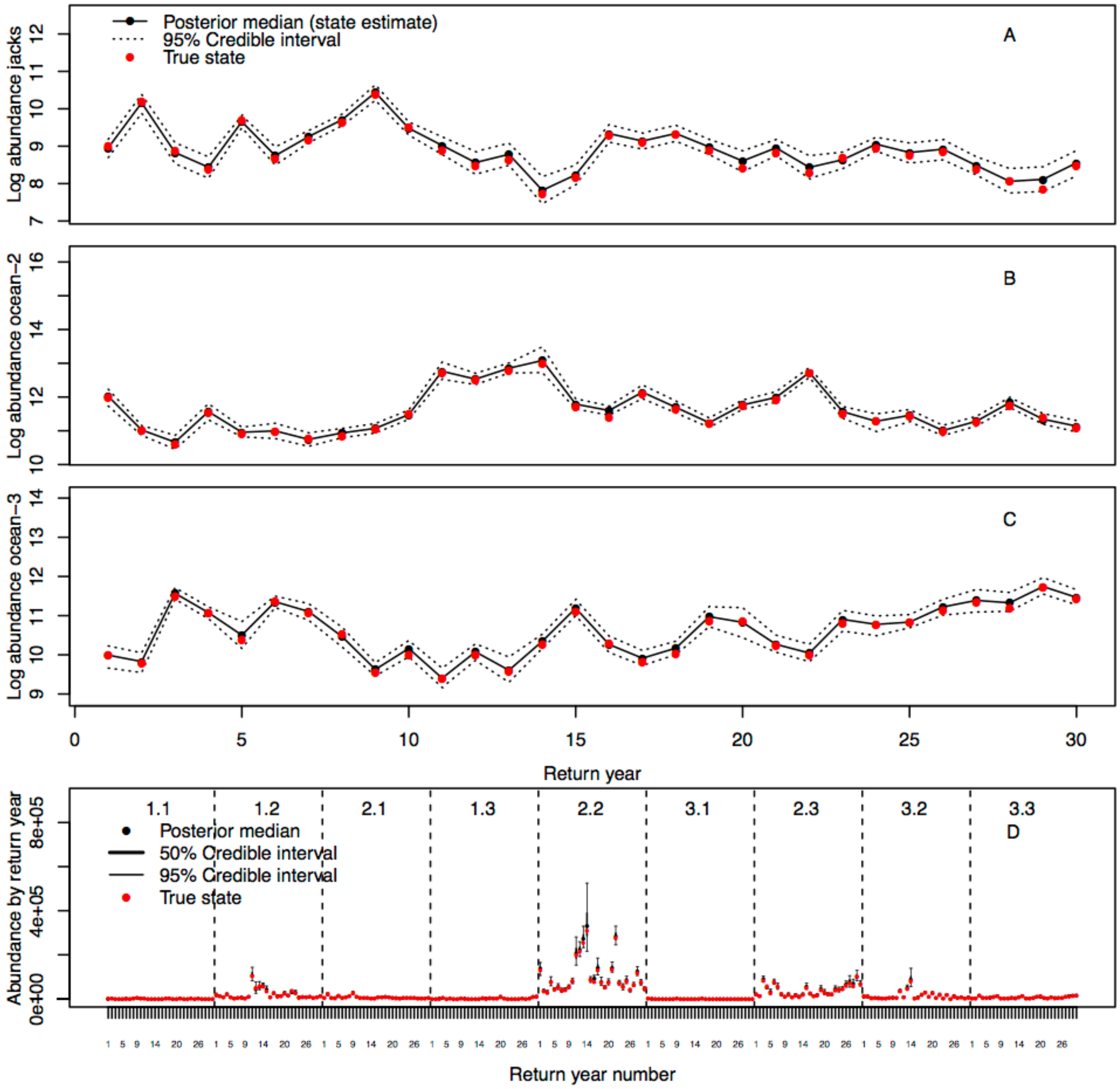
Posterior median (state estimate)
95% Credible interval
True state
A
Log abundance jacks
B
Log abundance ocean−2
C
Log abundance ocean−3
Return year
1.1
1.2
2.1
1.3
2.2
3.1
2.3
3.2
3.3
D
Posterior median
50% Credible interval
95% Credible interval
True state
Abundance by return year
Return year number

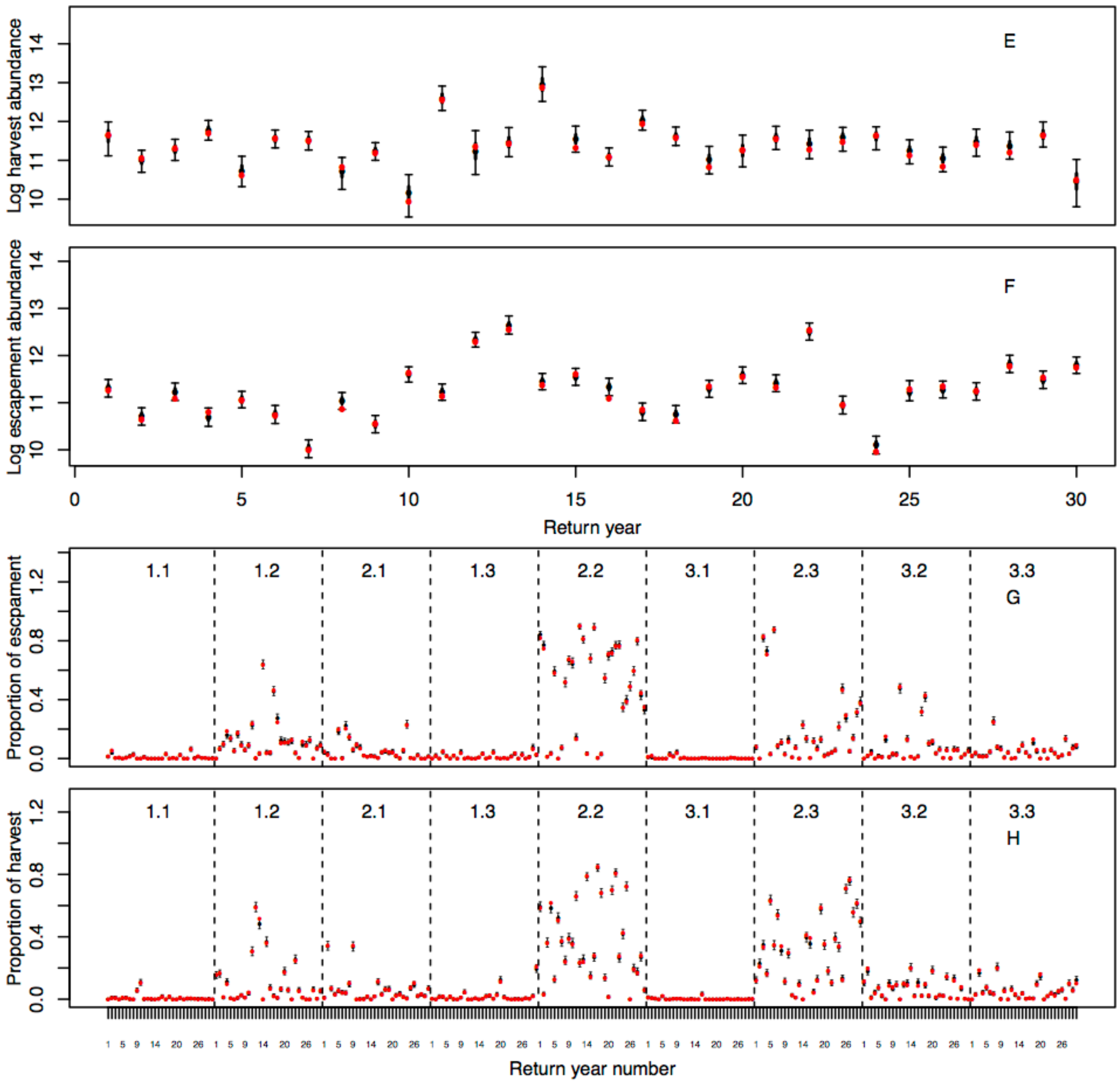

Figure S10. Model fit to simulated data for the multivariate model of return year abundance of ocean age classes (eq. 11). Panels A, B and C depict the credible intervals for state estimates of the return year log abundance of ocean age 1, 2, and 3 fish respectively relative to the values specified in the simulated data. Panel D depicts the credible intervals for estimated numbers-at-age by return year relative to the values specified in the simulated data. Panels E and F depict the credible intervals for the model-predicted state estimates of abundance ($\ln\sum_{a=1}^{\omega} R_{t,a}^{s}$) relative to the values specified in the simulation in the harvest and escapement respectively. Panels G and H depict the credible intervals for the model-predicted state estimates of age composition proportions ($\boldsymbol{\theta}_t^s$) over time relative to the values specified in the simulation in the escapement and harvest respectively.

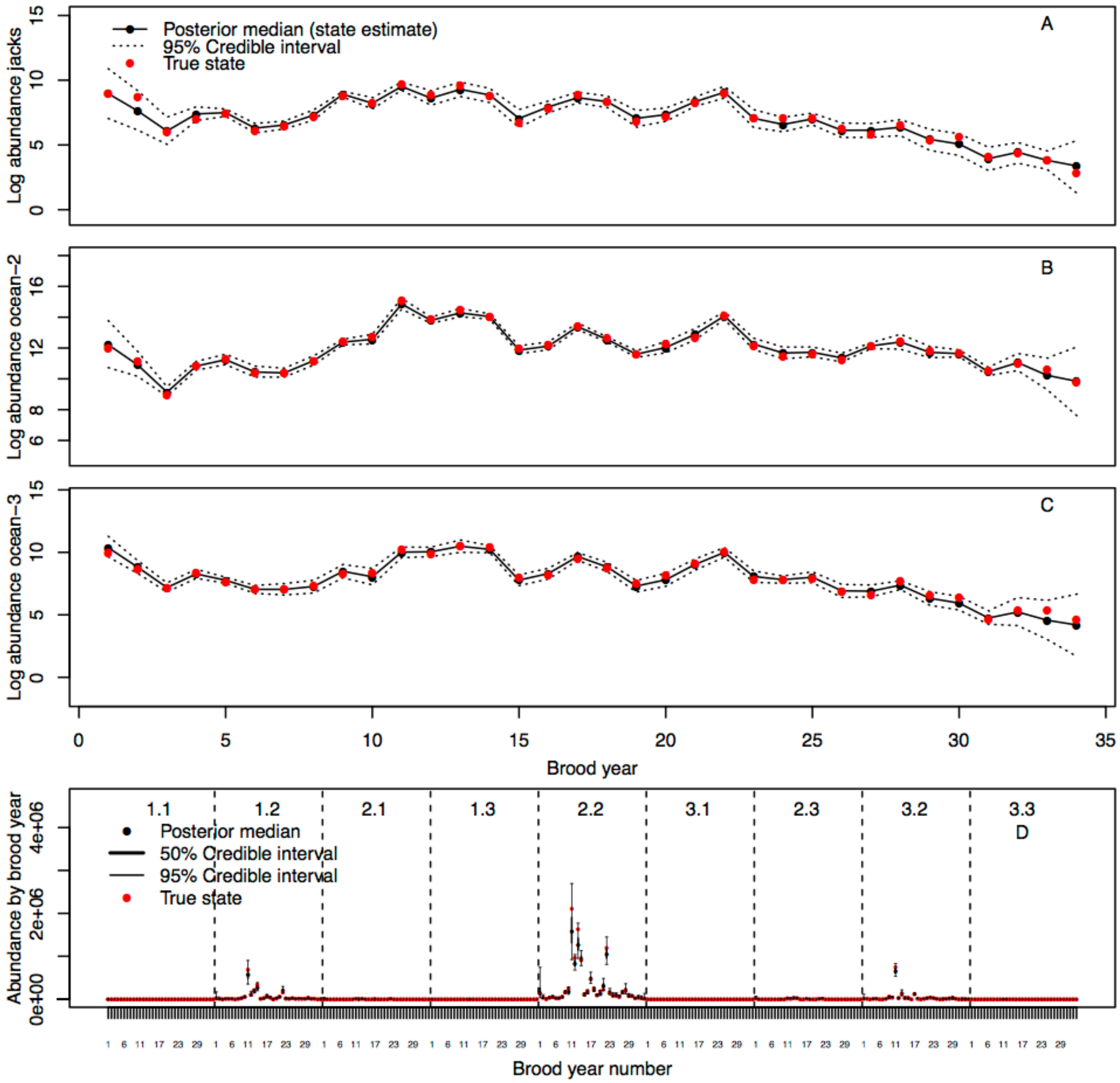
Posterior median (state estimate)
95% Credible interval
True state
A
Log abundance jacks
B
Log abundance ocean-2
C
Log abundance ocean-3
Brood year
1.1
1.2
2.1
1.3
2.2
3.1
2.3
3.2
3.3
D
Posterior median
50% Credible interval
95% Credible interval
True state
Abundance by brood year
Brood year number

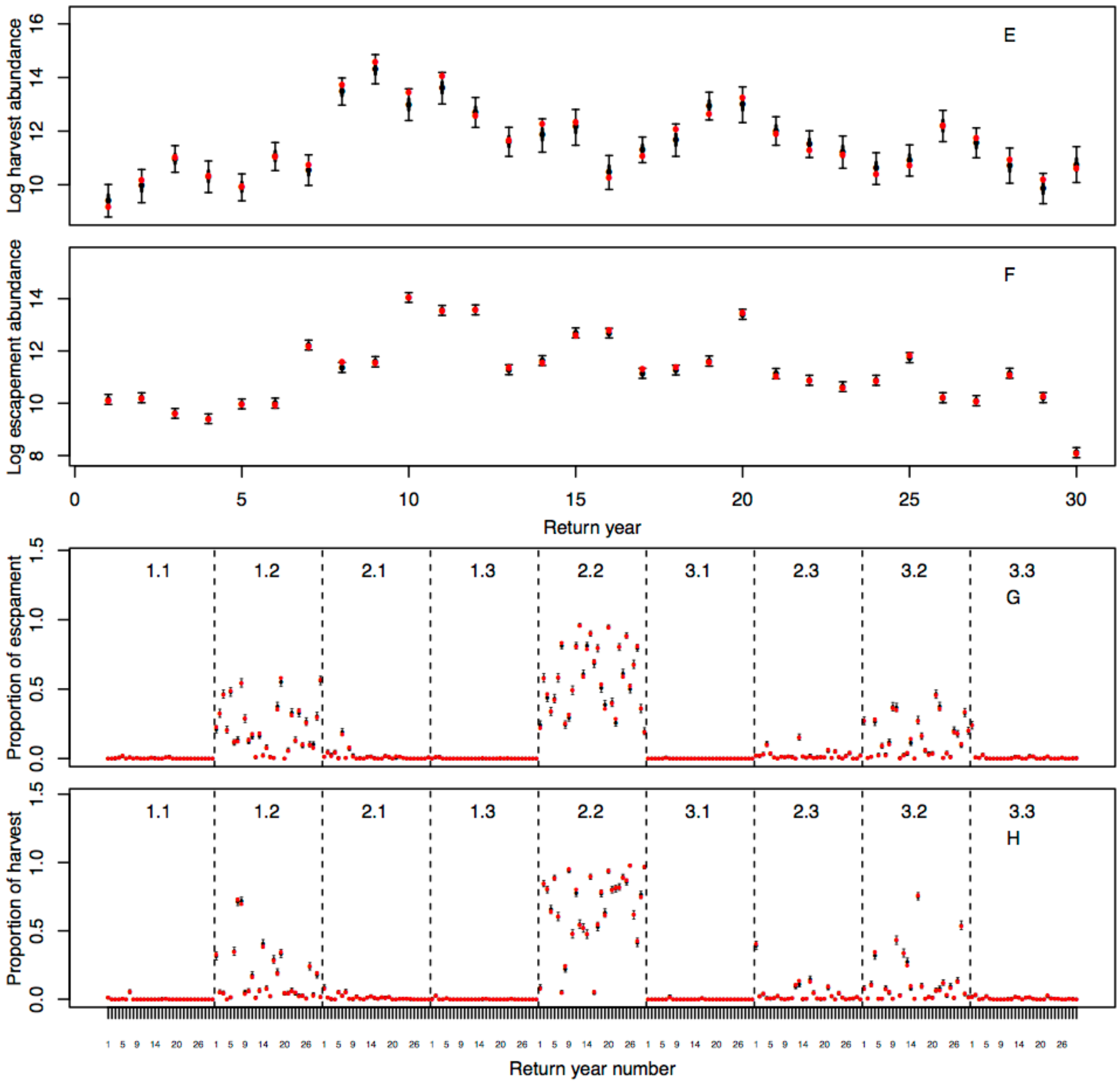

Figure S11. Model fit to simulated data for the multivariate model of brood year abundance of ocean age classes (eq. 10). Panels A, B and C depict the credible intervals for state estimates of the brood year log abundance of ocean age 1, 2, and 3 fish respectively relative to the values specified in the simulated data. Panel D depicts the credible intervals for estimated numbers at age by brood year relative to the values specified in the simulated data. Panels E and F depict the credible intervals for the model-predicted state estimates of abundance ($\ln \sum_{a=1}^{\omega} R^{s}_{t,a}$) relative to the values specified in the simulation in the harvest and escapement respectively. Panels G and H depict the credible intervals for the model-predicted state estimates of age composition proportions ($\boldsymbol{\theta}^{s}_{t}$) over time relative to the values specified in the simulation in the escapement and harvest respectively.

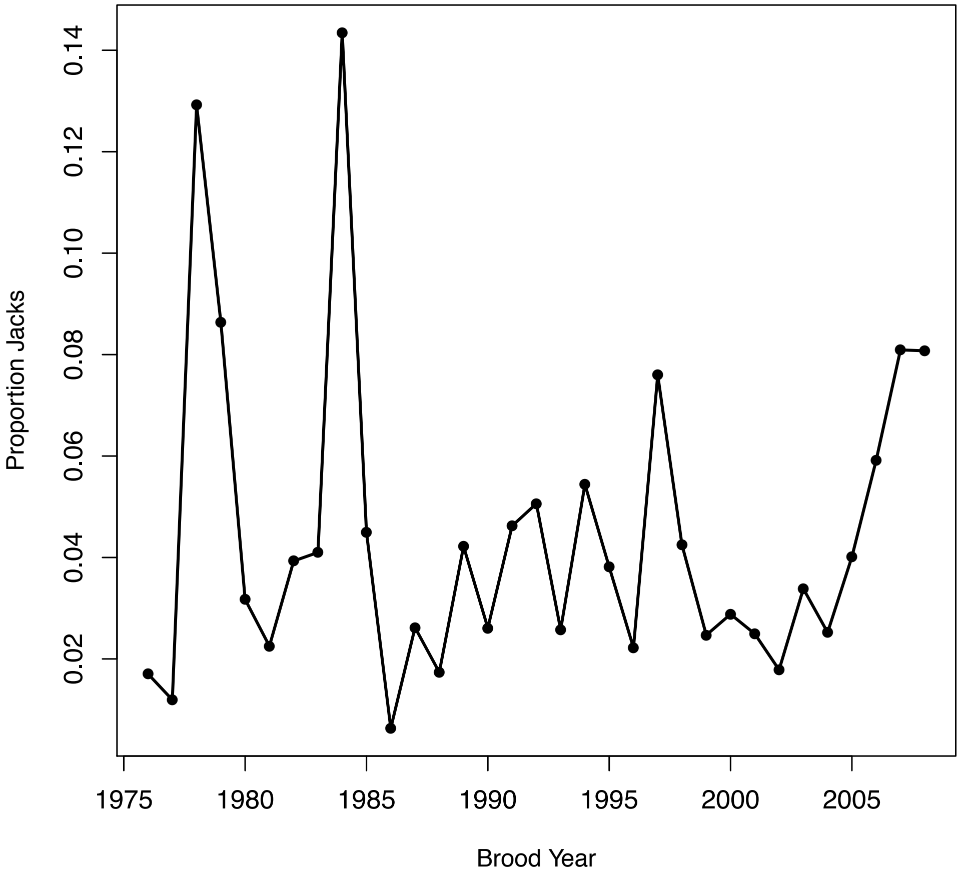

Figure S12. Jack proportions for the Ayakulik Sockeye salmon population by brood year over time.

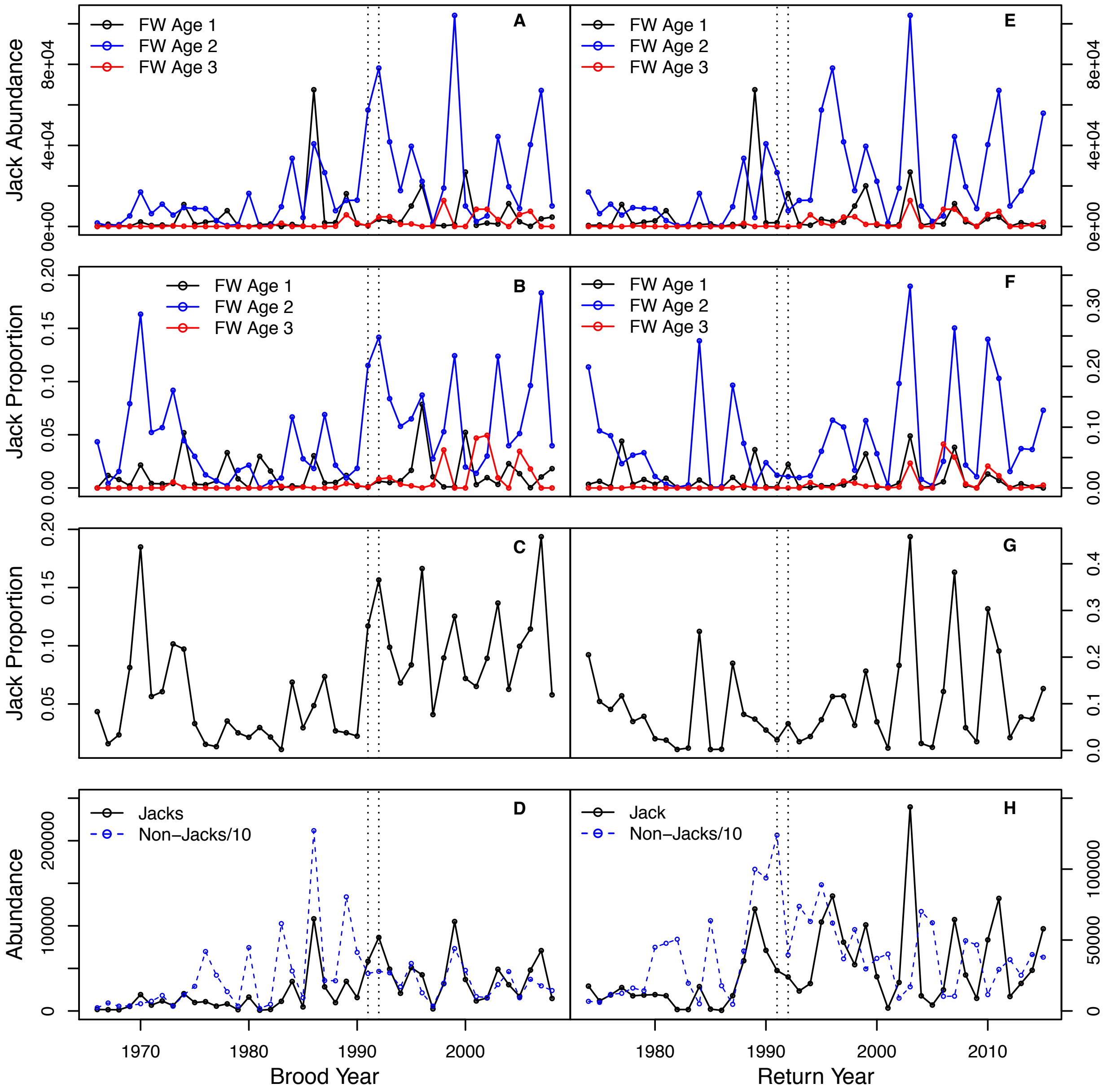

Figure S13. Age composition patterns over the full history of the Frazer Lake population (based on data from the ADF&G run reconstruction). Years 1991 and 1992 are indicated by vertical dotted lines. The 1991 run was dominated by ocean age-2 fish from the record-high 1986 recruitment, while most jacks spawning in this year were produced from the weaker 1987 cohort (Main text; Fig. 5B), leading to low jack proportions in that year's run (Main text; Fig. 5D). However, the 1987 cohort exhibited a relatively a high jack proportion (C). As such, although the 1991 run exhibited low jack proportions due to the numerical dominance of ocean age-2 fish, jacks were actually fairly abundant in this year's run (E). Importantly, 1991 was the first year in which the exploitation rate of jacks was substantially lower than that of older individuals (Main text; Fig. 6D) such that a large portion of the more abundant ocean age-2 and 3 fish were harvested, leaving a high proportion of jacks in the spawning escapement (Main text; Fig. 3A). Jacks were also a large portion of the spawners in 1992, partially due to continued higher exploitation rates on older fish in this year as well (Main text; Fig. 6D). Additionally, while most

Frazer Lake sockeye mature at freshwater age-2, a large portion of jacks from the strong 1989 cohort spent only one year in freshwater for unknown reasons (A). These abundant three-year-old jacks returned to spawn in 1992, among ocean age-2 fish from the weak 1987 recruitment (Main text; Figure 6B).

Table S1. Index of models, associated equations, and data.

Process model	Other equations	Associated figures	Data used
Eq. 1	N/A	Fig. 2	ADF&G run reconstruction: There is no record of scale sample sizes prior to 1986, and thus no information on the uncertainty in the age composition likelihoods for a state-space model. However, abundance-at-age based on deterministic calculations of age composition and abundance (not model output, no propagation of uncertainty) are available going back to brood year 1968. To estimate long term trends in jack proportions over the longest time period possible, these values are used in eq. 1 but we use the raw scale and abundance data in subsequent models.
Eq. 2	Eq. 3-9, 14,15	Figs. 3, 4	Numbers-at-age in the scale samples, and abundance for harvest and escapements
Eq. 10	Eq. 6 (modified), 7-9, 12, 14, 15	Fig. 5A-C	Numbers-at-age in the scale samples, and abundance for harvest and escapements
Eq. 11	Eq. 6 (modified), 8, 9, 13-15	Figs. 5D-F, 6	Numbers-at-age in the scale samples, and abundance for harvest and escapements
